# Design of lightweight metal surface defect detection technology for YOLOv7-tiny using Anchor-Free algorithm

**DOI:** 10.1038/s41598-026-39233-9

**Published:** 2026-02-13

**Authors:** Yi-Cheng Huang, Jun-Chang Lin, Yi-Ze Wu

**Affiliations:** Department of Mechanical Engineering, National Chung Hsing University, Taichung, 402202 Taiwan

**Keywords:** Deep learning, Metal surface defect detection, YOLOv7, Anchor-Free method, MobileNetV3, Attention mechanism, Engineering, Mathematics and computing

## Abstract

Accurate identification of defect morphology and size distribution on metallic surfaces is crucial for manufacturing, particularly in the steel industry where such materials are widely used. This study proposes a novel lightweight architecture based on YOLOv7-tiny to address the challenges of real-time metallic surface defect detection. The original anchor-based detection head is replaced with an anchor-free mechanism to reduce missed detections of defects with extreme aspect ratios, while a logarithmic transformation–based enhancement is introduced to strengthen defect features. The backbone is replaced by the lightweight MobileNetV3-large network to reduce the number of parameters, and an Efficient Multi-Scale Attention (EMA) module is embedded into the bottleneck structure to increase target importance and suppress background interference, especially for small defects. In addition, a bidirectional feature pyramid network with adaptively spatial feature fusion (BAFPN) is integrated as the Head architecture to provide richer semantic information. The improved model was evaluated on the DAGM 2007, NEU-DET, and GC10-DET datasets, which encompass metal surface defects with varying degrees of complexity. The results demonstrate an average increase of 6.24% in mean average precision (mAP) and an inference speed over 90 FPS, confirming both the efficacy and real-time capability of the proposed method.

Steel plates are a fundamental raw material for many industries and are extensively employed throughout industrial production and manufacturing processes. For example, in the automotive industry, steel plates are used to fabricate vehicle bodies and components, whereas in the construction industry they are used to build structural frames and support structures. However, due to limitations in manufacturing conditions, defects on steel surfaces are unavoidable, such as cracks, scratches, and inclusions. These defects directly affect product performance, including corrosion resistance, wear resistance, and fatigue strength, degrade the visual quality and safety of the product, and shorten its service life. The surface quality of steel plates has become a critical indicator in determining their price, and assessing the severity of surface defects is of great significance for the economic performance of steel manufacturers. Consequently, surface defect inspection has become an indispensable stage in the steel plate production process.

With the advancement of smart manufacturing, deep learning–based object detection technologies have attracted considerable attention across manufacturing industries due to their strong representation learning capability and high efficiency. A variety of algorithms and network architectures have been proposed and widely deployed in different fields, including agriculture^1^, healthcare^2^, and the automotive industry^3^. Among the numerous object detection algorithms, YOLO (You Only Look Once)^4^ has emerged as a particularly popular approach in recent years owing to its real-time performance, high efficiency, and the rapid progress of related research.

Zhang^5^ et al. investigated automated automotive paint defect detection and proposed a novel data augmentation strategy that generates additional samples by performing random sampling around target regions with an intersection over union (IoU) of at least 25%. They further introduced an improved SSD-based object detection algorithm, MobileNet-SSD, which employs a modified MobileNet as the backbone network of the Single Shot MultiBox Detector (SSD) framework. On their self-constructed automotive paint defect dataset, the proposed method achieved a detection accuracy of 95%. However, in the conclusion, the authors noted that when predicting small objects from low-level feature maps, the model suffers from insufficient semantic information from high-level feature maps, resulting in suboptimal recognition accuracy for small-sized defects. This observation indicates that existing methods still face challenges and leave room for improvement in the detection of small objects. Wang^6^ and Cheung proposed a novel model, CenterNet-CL, based on CenterNet and augmented with a counting loss, for defect detection in additive manufacturing (AM) of optical components. They added an extra prediction head dedicated to density map estimation and counting loss. Compared with the conventional CenterNet al.gorithm, the new model can simultaneously extract the type, location, and quantity of defects while improving overall detection performance. Chen^7^ et al. enhanced the Faster R-CNN model to improve defect detection in textile products. They introduced Gabor filter kernels into the first layer of the Faster R-CNN network and employed a genetic algorithm (GA) to select the optimal filter parameters, thereby leveraging the strong texture analysis capability of Gabor filters to suppress interference from background textures. In addition, ResNet50 was adopted as the backbone network within the Faster R-CNN framework to strengthen the extraction of high-dimensional features. The proposed method achieved a detection accuracy of 94.57% on a textile defect dataset containing 6,316 images. Pan^8^ et al. proposed an image enhancement algorithm for low-contrast X-ray images, termed GCE, together with an improved YOLOv5-based model called WD-YOLO for weld defect detection. Their study outlined three major challenges: (1) highly complex backgrounds in weld images, (2) the lack of clear boundaries between defects and background, and (3) significant size variation across different types of welds. The GCE algorithm removes image artifacts based on pixel distribution and image resolution and performs adaptive gray-level stretching according to the grayscale curve. The WD-YOLO model adopts a new backbone architecture, NeXt, to enhance feature extraction. Furthermore, a bi-level routing attention (BRA) module is incorporated into the small-object detection layer to strengthen the model’s capability in detecting tiny defects. Ultimately, WD-YOLO achieved a detection accuracy of 92.6% at 98 frames per second on a self-constructed weld defect dataset.

In recent studies, Shen^9^ et al. adopted a cross-modal interaction (CMI) strategy for metallic surface defect detection, integrating information from both vision and language modalities so that the model can retain strong generalization capability while still detecting specific defects. They proposed a vision–language cyclic interaction model (VLCIM), which combines multi-domain feature extraction, cyclic interaction learning, and domain-specific design. On a public control valve core dataset, this method improved detection performance by 5.4% and achieved an average precision of 88.5% for each defect category. In a related work, Shen^10^ et al. also addressed the strong interference caused by defect variability and background gray levels in detecting surface defects on control valve core. They proposed a multi-expert diffusion model comprising a multi-feature extraction module, a low-pass guided feature aggregation module, and a heterogeneous diffusion detection mechanism. This multi-expert model achieved an average recall of 82.6% on a public dataset of control valve core surface defects.

The above literature demonstrates that optimization methods based on architectural and model-engineering modifications are highly effective in enhancing model performance. At the same time, combining such approaches with image processing techniques—such as data augmentation, feature enhancement, and texture analysis—offers substantial advantages for defect detection in complex scenes with high-dimensional features. Nevertheless, despite the rapid and diversified development of object detection algorithms, applying them to metallic surface defect inspection still presents several difficulties and challenges: (1) real-time inspection in industrial applications must simultaneously satisfy requirements on detection speed and accuracy, so accuracy must be maximized under constrained computational resources; (2) the complex backgrounds and weak or missing boundaries of metallic surface defects pose a significant challenge to detection accuracy; and (3) the wide variation in defect size, including extreme scale differences and very small defects, further increases the difficulty of reliable defect recognition.

Therefore, developing a general-purpose system capable of real-time and accurate detection of various types of defects is an extremely challenging task. To address the aforementioned difficulties, this study adopts the fast and robust YOLO family of models, and selects YOLOv7-tiny^11^ as the primary backbone architecture to ensure detection speed. To further accelerate inference, we incorporate MobileNetV3 to redesign and lightweight the backbone of YOLOv7-tiny. To cope with the complex backgrounds of metal surfaces, we embed a bidirectional feature pyramid network with adaptively spatial feature fusion (BAFPN) into the network architecture to enhance feature integration with merely increasing computational cost, and apply a logarithmic transformation–based algorithm for contrast enhancement of the image data. Finally, to handle the large variation in defect sizes and the presence of very small defects, we employ an Anchor-Free detection head and the Efficient Multi-Scale Attention (EMA) attention module. The former relaxes the constraints imposed by predefined anchor sizes and improves the detection of defects with extreme scale variation, while the latter increases the attention to small objects and thus enhances their discriminability. In summary, the main contributions of this work are as follows:

1. We propose a novel lightweight metal surface defect detection model based on the YOLOv7-tiny architecture, which satisfies the real-time detection requirements of modern industrial applications while effectively addressing complex and hard-to-distinguish defects and maintaining high accuracy.
2. We introduce a log transformation–based feature enhancement scheme that strengthens the representation of metal surface defects and improves their recognizability.
3. Using the proposed model, we conduct ablation studies, comparative experiments, and test-set evaluations on three public metal surface defect datasets to clarify the impact of each optimization component and to validate the effectiveness and generalization capability of the proposed approach.

## Methods

### Datasets

This study selected three publicly available metal surface defect datasets with different complexity levels as experimental subjects to verify the effectiveness and generalization of the proposed method. These publicly available datasets are widely recognized, with their rigor and reliability verified.

#### DAGM 2007 dataset

**Fig. 1:**
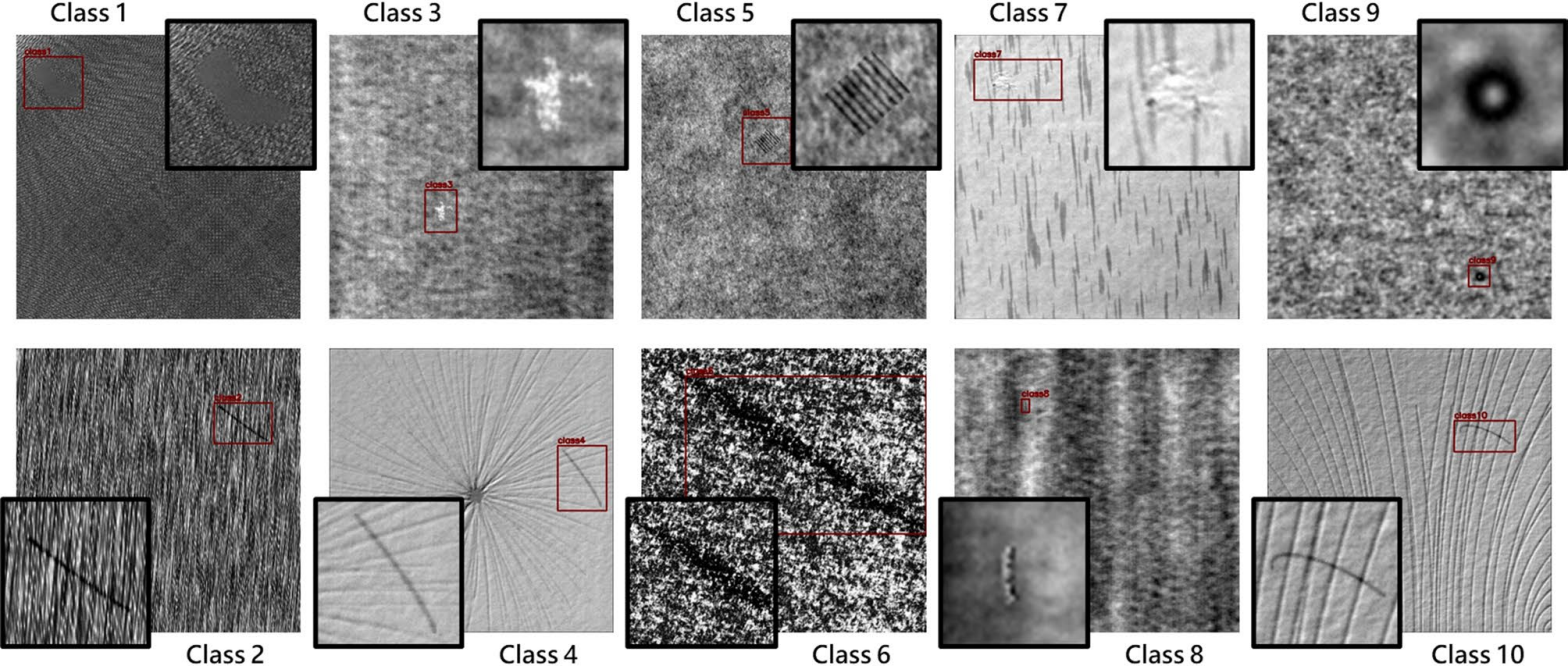
Examples from the DAGM 2007 dataset.

The DAGM 2007 dataset^12^ is an industrial optical inspection weakly supervised learning competition topic proposed by the German DAGM symposium in 2007. The images are artificially synthesized industrial surface textures with 512 × 512 pixels grayscale images, and the defects are divided into ten categories, as shown in Fig. 1 Since the proportion of non-defective data in the DAGM 2007 dataset is the majority (87%), this study removes all non-defective data to avoid excessive negative sample training, leaving a total of 2100 images as the low-complexity dataset for this study.

#### NEU-DET dataset

**Fig. 2:**
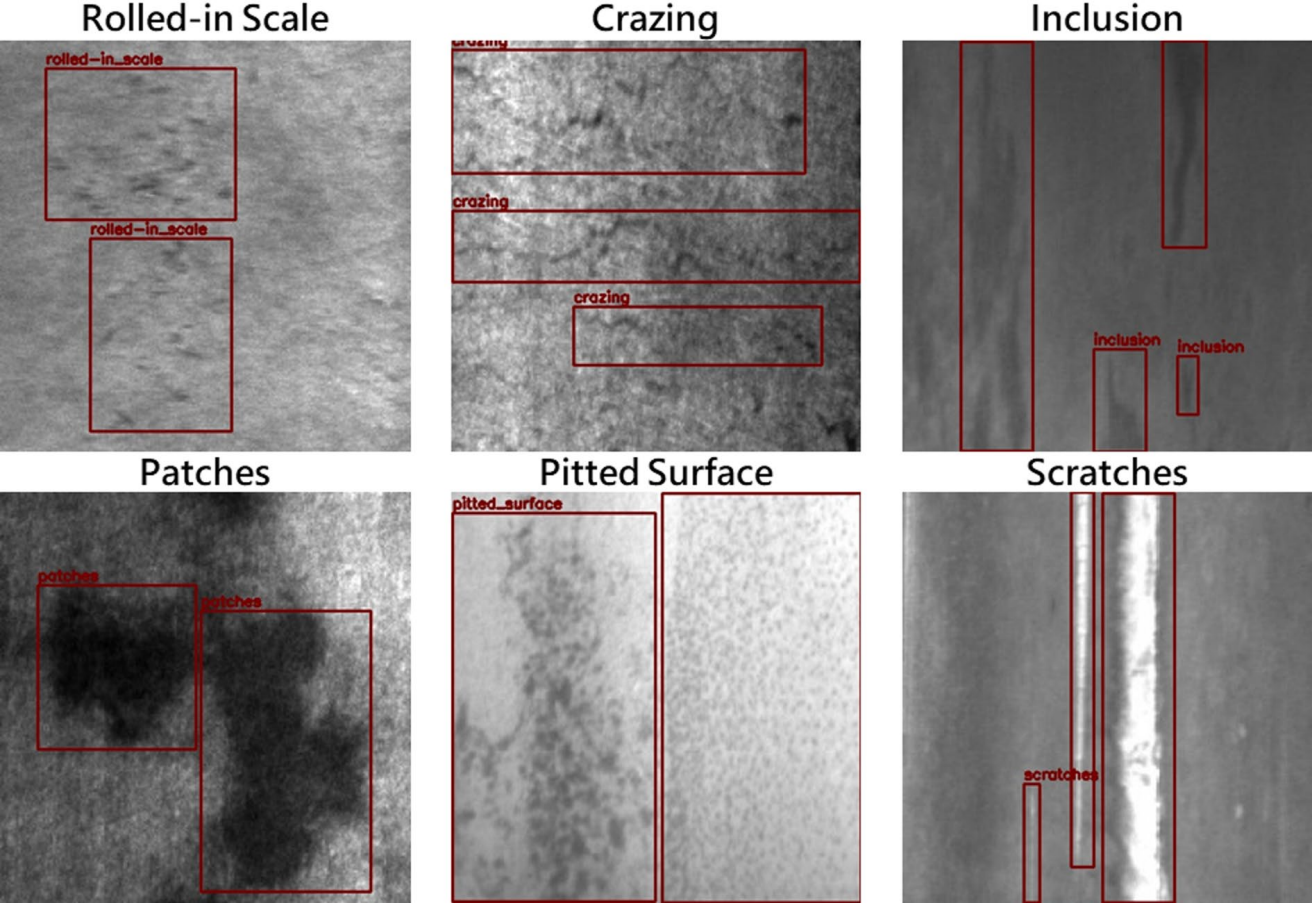
Examples from the NEU-DET dataset.

The NEU-DET dataset^13^ is a hot-rolled steel strip defect dataset proposed by He et al. from Northeastern University in China. The images are 200 × 200 pixels grayscale images, and the dataset collects six typical defects of hot-rolled steel strips, including Rolled-in Scale, Patches, Crazing, Pitted Surface, Inclusion, and Scratches, as shown in Fig. 2. Each category contains 300 images, totaling 1799 images, as the medium-complexity dataset for this study.

#### GC10-DET dataset

**Fig. 3:**
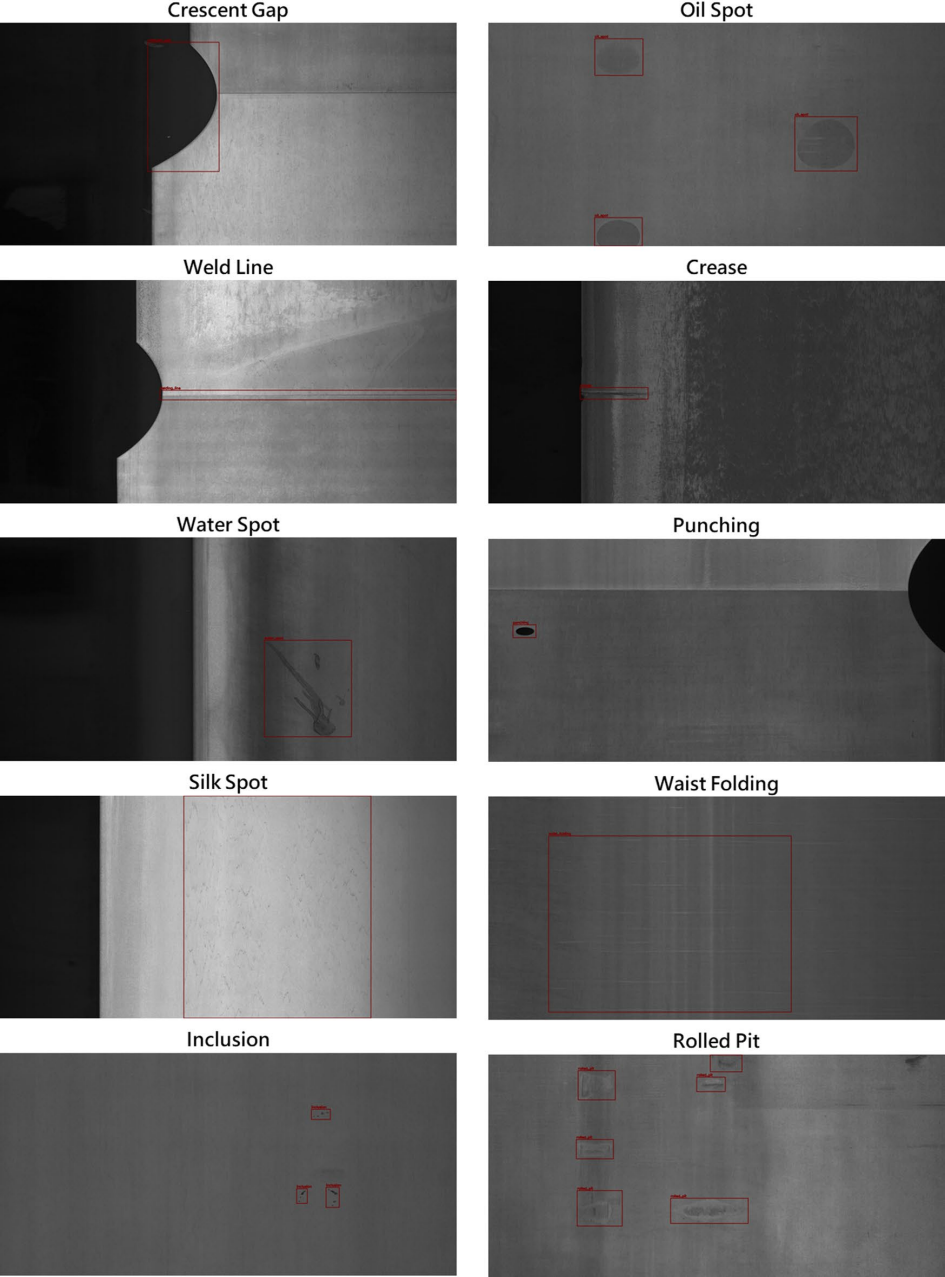
Examples from the GC10-DET dataset.

The GC10-DET dataset^14^ is a real industry steel strip defect dataset proposed by Lv et al. from Tianjin University in China to address the current lack of research data scale and limited defect categories for metal surface defects. The images are 2048 × 1000 pixels grayscale images, covering ten types of surface defects, including Crescent Gap, Weld Line, Water Spot, Silk Spot, Inclusion, Oil Spot, Crease, Punching, Waist Folding, and Rolled Pit, as shown in Fig. 3. The dataset scale offered 3570 images, with only 2294 images publicly available as the dataset in this study.

### Model and datasets improvement

Compared to other YOLO versions, YOLOv7 introduces an Efficient Layer Aggregation Networks (ELAN) architecture, which analyzes the gradient path to enable deeper networks to effectively learn and converge. For training, it adopted methods from the Bag-of-Freebies approach, such as Model Re-Parameterization and Dynamic Label Assignment Strategy, effectively improving the model performance and efficiency.

**Fig. 4:**
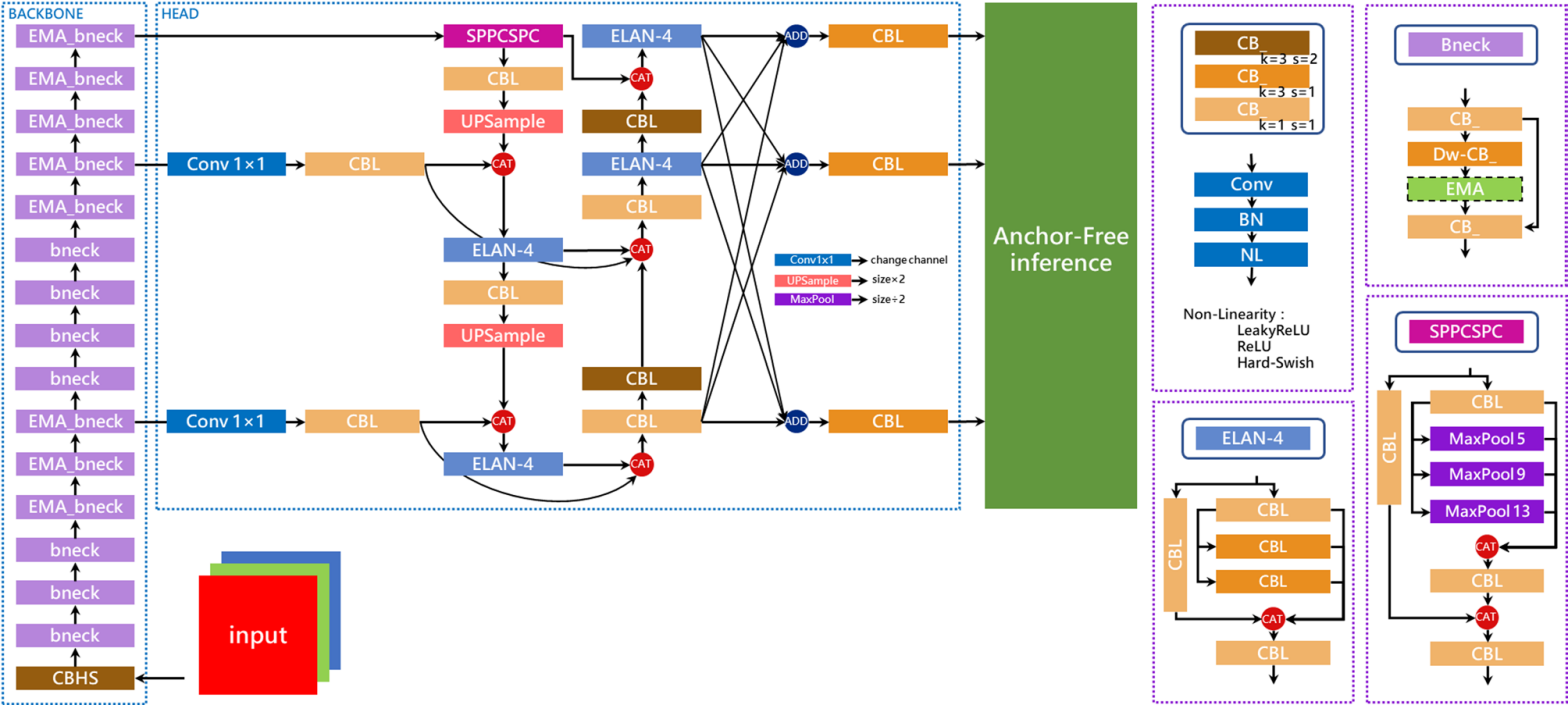
Improved network model architecture based on YOLOv7-tiny.

However, despite the many advantages of YOLOv7, applying it to real-time defect detection with high complexity and high-dimensional features still faces many challenges. Therefore, in this study, we propose an improved lightweight model architecture based on the YOLOv7-tiny model, as shown in Fig. 4, to address the challenges faced in real-time metal surface defect detection in modern industries. The main network structure improvements include the following parts.

#### Anchor-Free detection head

Since YOLOv2, the detection head concept of the YOLO series object detection algorithms has transitioned from Anchor-Free to Anchor-Base to optimize the algorithm. The Anchor-Base method uses the k-means algorithm to fit a set of predefined boxes (Anchor Box) that best match the dataset, allowing subsequent predictions to fine-tune the offset of the Anchor Box to accurately locate the target position. Compared to directly predicting the boundary box (Predicted Box), it simplifies the prediction problem to some extent, making the model easier to learn. Additionally, predefined boxes have a strong constraint effect, enabling higher matching accuracy and improving recognition effects.

However, with the development and enhancement of neural network technology, the learning ability of models has significantly improved, bringing renewed attention to the Anchor-Free method for directly predicting the Predicted Box. Conversely, the constraints executed by the Anchor-Base method have caused negative effects when dealing with extreme targets in images. For instance, in cases of targets with extreme aspect ratios or sizes, the fitted Anchor Boxes may be difficult to cover and may even result in detection omissions.

**Fig. 5:**
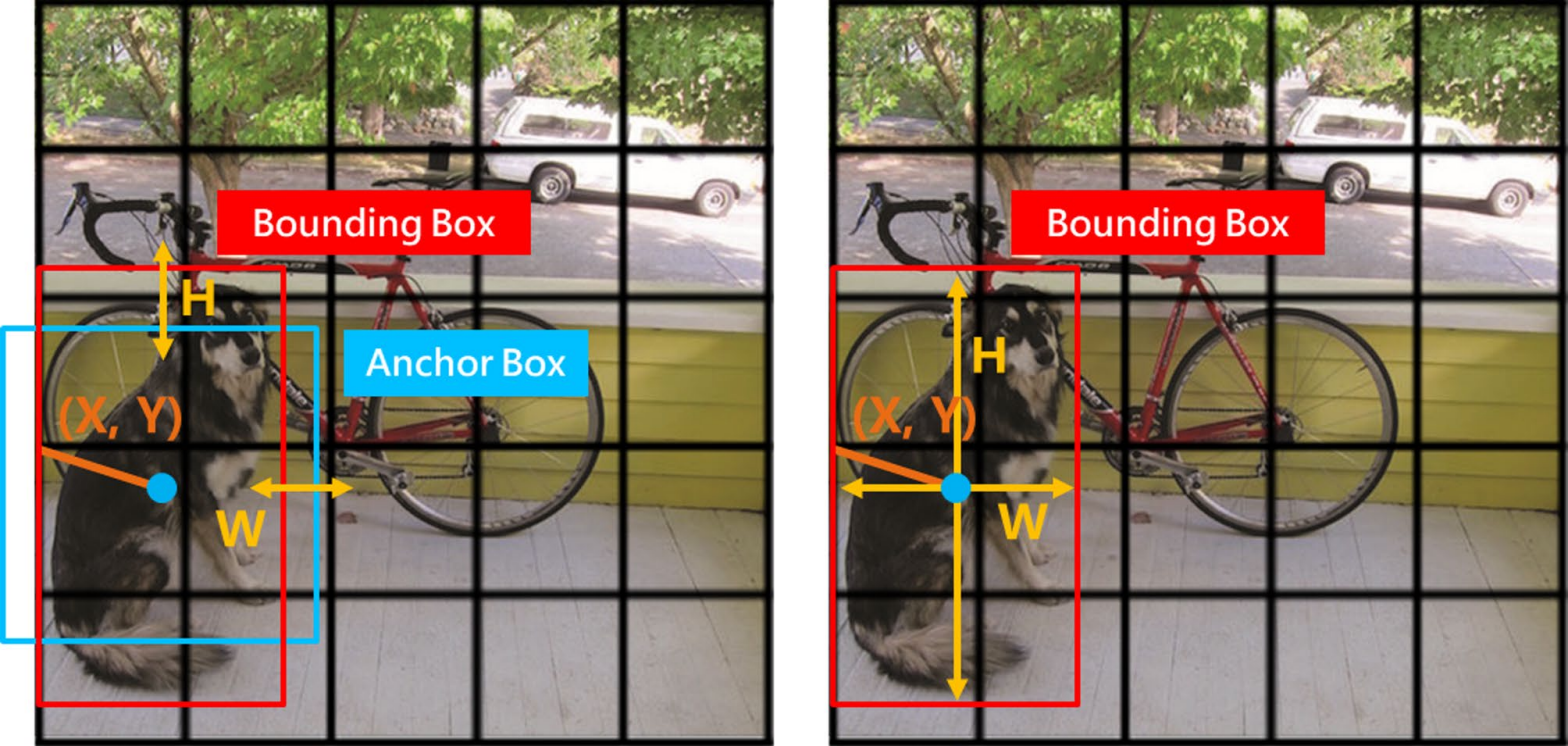
Difference between Anchor-Base(left) and Anchor-Free(right) detection heads.

To address the problem of detecting extreme size defects on metal surfaces caused by the Anchor-Base method in YOLOv7, this study adopts the Anchor-Free detection head from YOLOv9^15^, developed by the same authors and scheduled for pre-publication in 2024. This detection head employs a center-based method, which first identifies the central area and then predicts the distances from the center point to the four boundaries to determine the Predicted Box position, as illustrated in Fig. 5.

#### Dataset annotation optimization

**Fig. 6:**
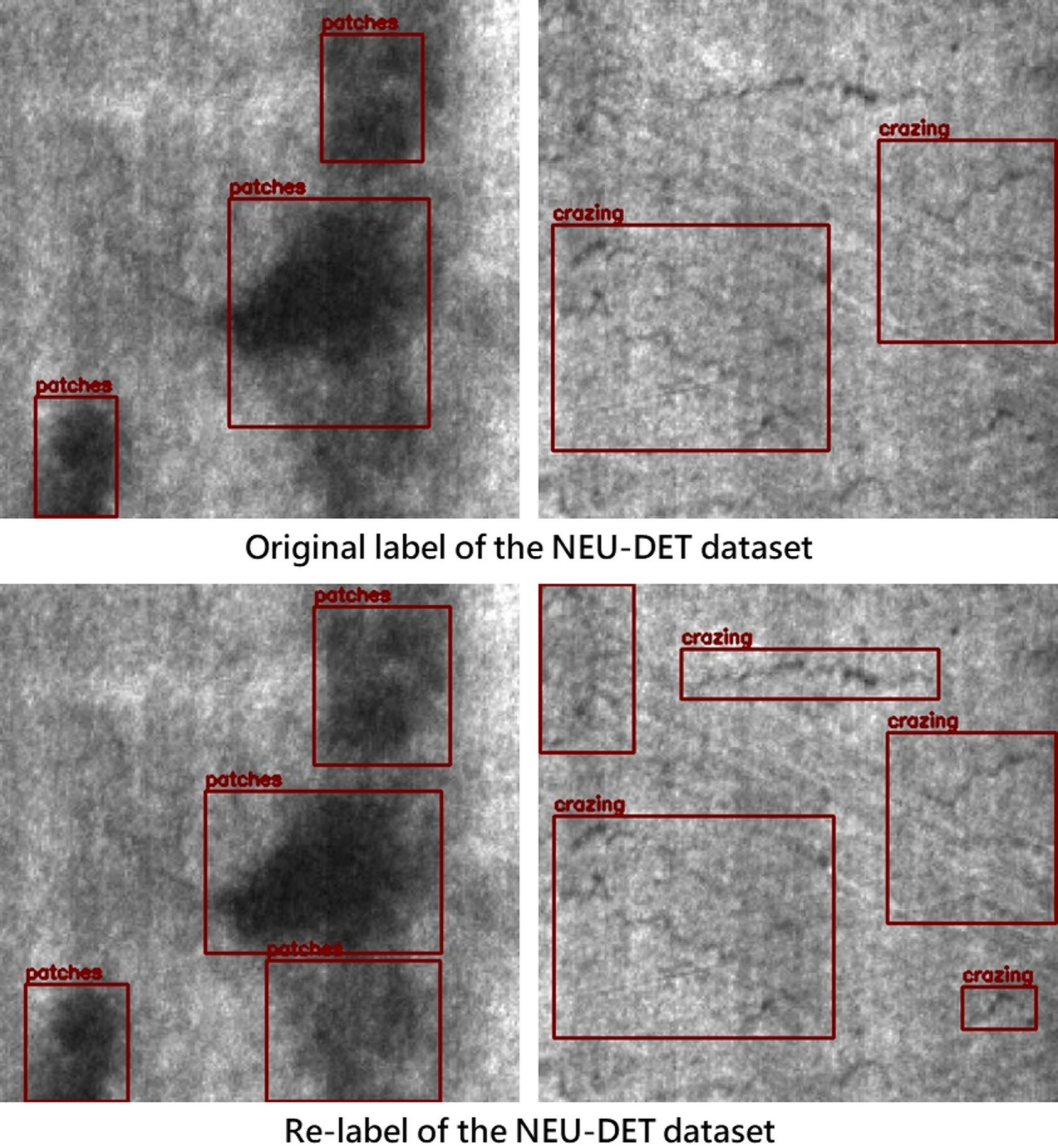
Re-annotation results for NEU-DET dataset.

**Fig. 7:**
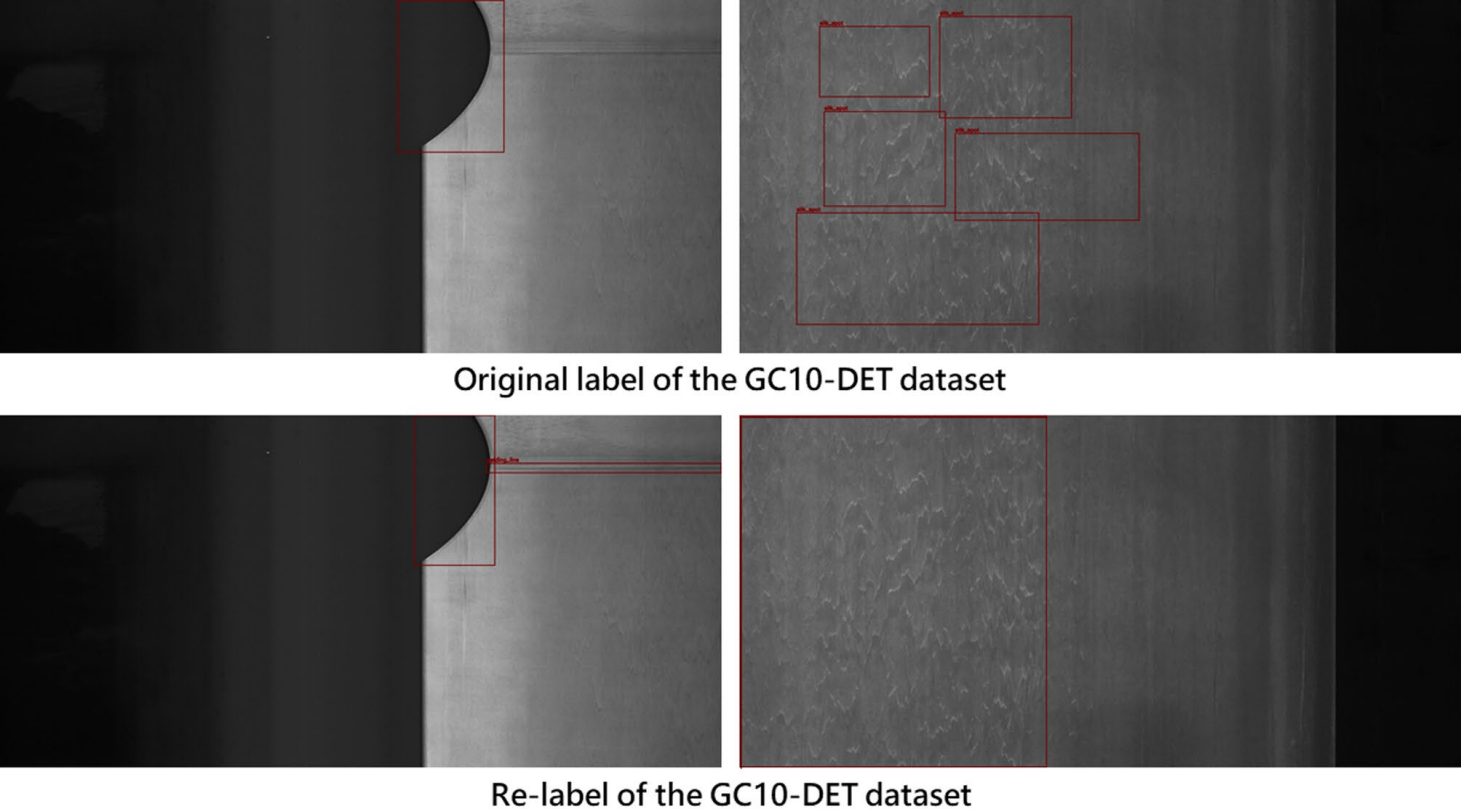
Re-annotation results for the GC10-DET dataset.

The quality of the dataset significantly affects the accuracy of object detection. No matter how advanced the model or algorithm, if the dataset’s annotations are inaccurate or erroneous, the accuracy will be limited by the dataset’s quality. Through observation, this study found that there is still room for improvement in the annotation accuracy of the NEU-DET and GC10-DET public datasets. Therefore, we re-annotated both datasets according to the provided categories, with the results shown in Figs. 6 and 7.

To maintain consistency in re-annotation as much as possible, this study exclusively utilized a single annotator to complete the re-labeling of the entire dataset. This was done to mitigate bias resulting from inter-annotator variability in labeling standards. In addition, to ensure a consistent basis for visual judgment, this relabeling step was performed prior to the image feature enhancement procedures. As shown in Figs. 6 and 7, the total number of labels might increase or decrease after relabeling. This change primarily results from our optimization of the original annotations through supplementation, contraction, and merging under the following conditions: (1) defects extending beyond the original bounding box, where the adjustment criterion is the presence, outside the bounding box, of texture or intensity patterns similar to those inside the box; (2) bounding boxes that include excessive background, where the adjustment criterion is the presence, within the box, of defect-free surface texture similar to the good regions outside the box; and (3) missing annotations, where the judgment is based on defect textures or patches appearing in other regions of the same image or in other images— in the former case reflecting that identical defects tend to occur in similar areas, and in the latter indicating that certain defects were originally left unlabeled.

In summary, using a single annotator in combination with the above relabeling criteria improves the overall consistency of the dataset annotations, mitigates variability caused by subjective labeling standards, and reduces the likelihood of missed defects. Furthermore, this study also merges labels in cases where two or more bounding boxes are placed on defects that are extremely close to each other or in fact correspond to a single defect instance. Such over-segmentation is likely a consequence of earlier studies having to satisfy size and aspect-ratio constraints imposed by anchor-based detection frameworks. Since the detection model adopted in this work employs an anchor-free detection head, these labels are merged to better approximate the actual morphology and extent of the defects.

**Fig. 8:**
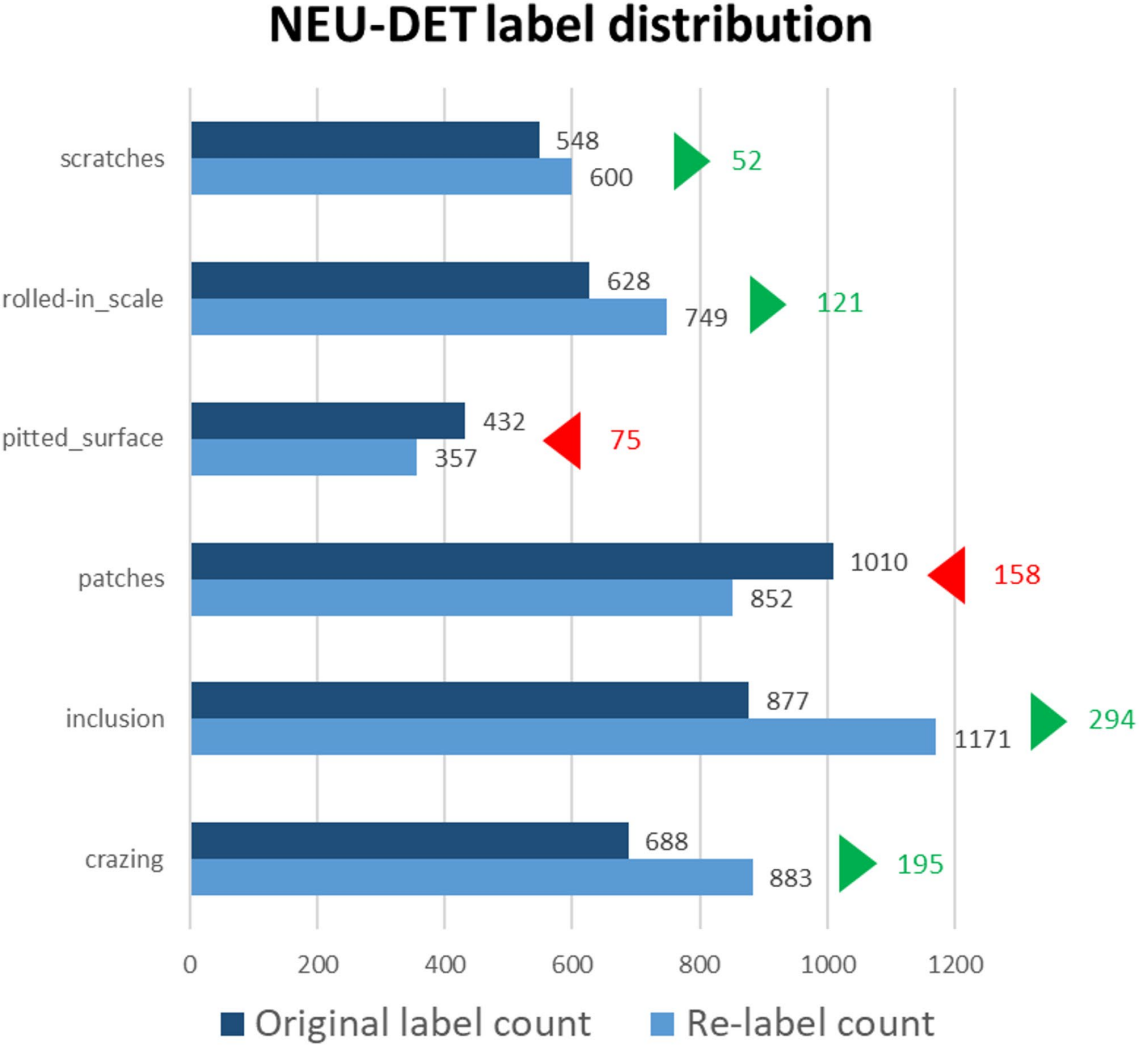
Label distribution and quantity changes in NEU-DET.

**Fig. 9:**
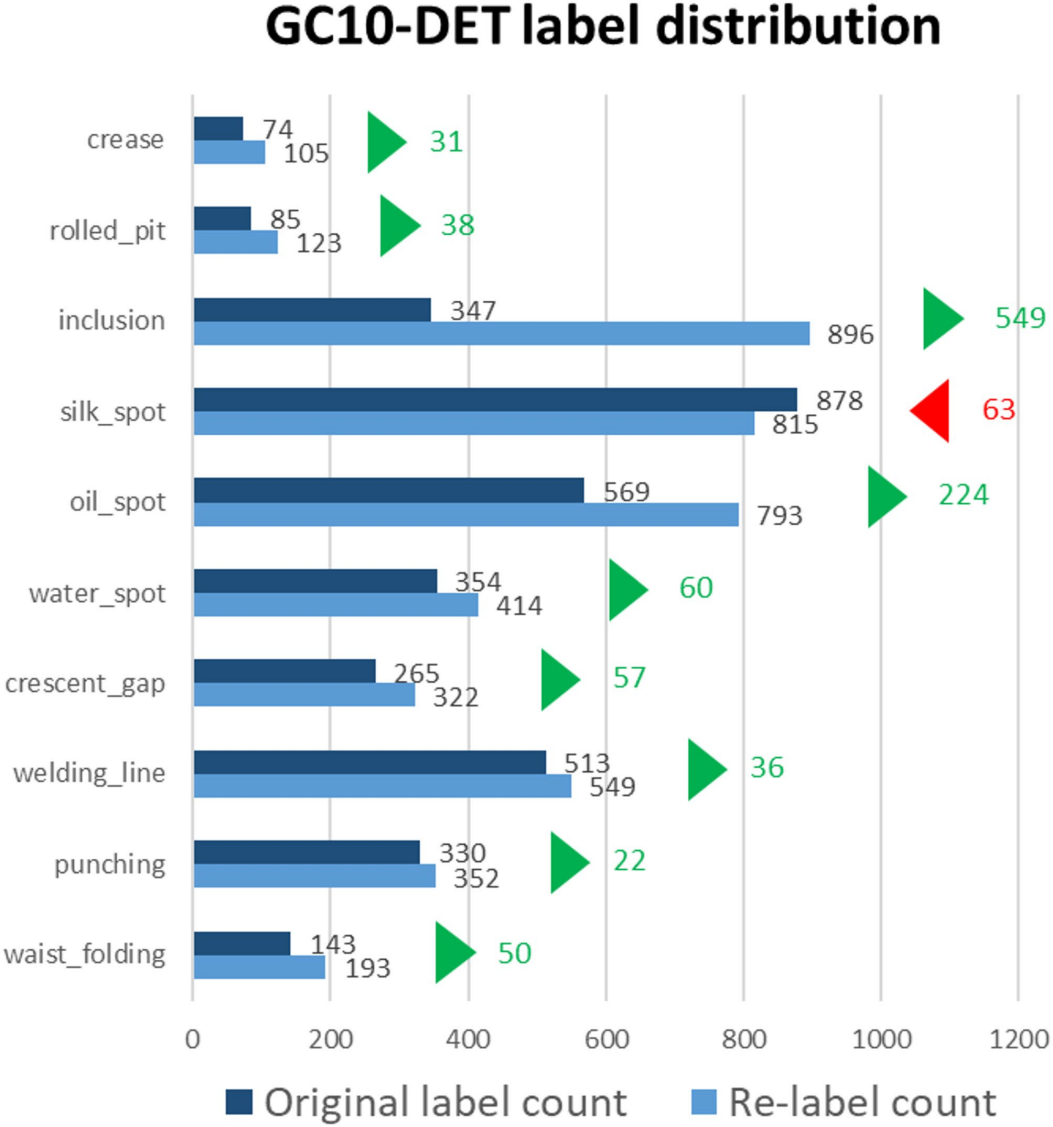
Label distribution and quantity changes in GC10-DET.

To ensure the rationality of the rebuilt dataset in this study, we conducted a statistical analysis of the changes in the number of labels. As shown in Figs. 8 and 9, the results indicate that the number of defect labels increased for most categories after re-annotation.

#### Feature enhancement

**Fig. 10:**
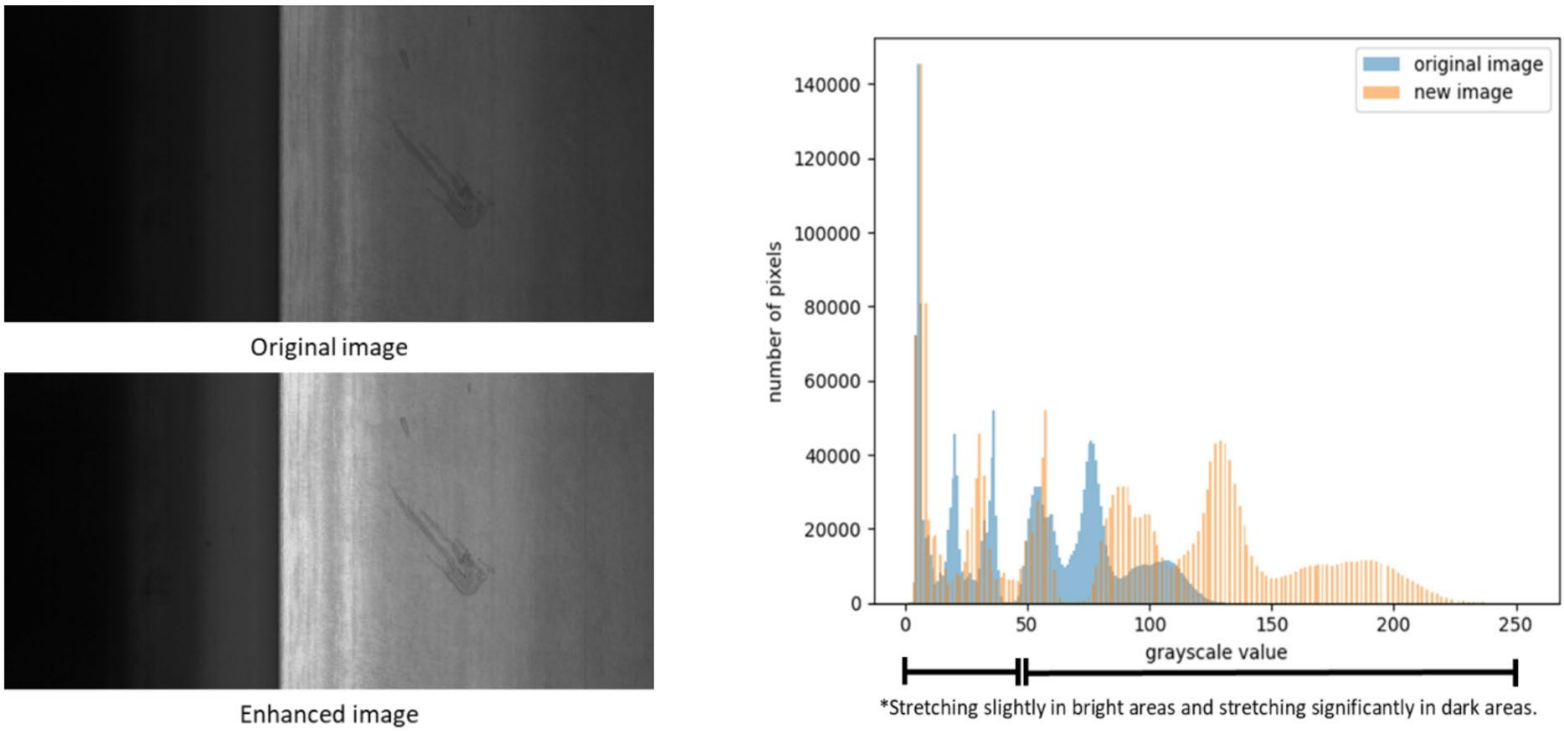
Illustration of the feature enhancement effects.

In the inspection of metal surface defects, certain defect textures are extremely subtle and indistinct, and are easily obscured by background patterns. Therefore, appropriate image processing techniques are required to enhance defect features and thereby effectively separate defects from the background. The fundamental difficulty lies in the high similarity of tone and intensity between the defects and the surrounding metallic texture. In grayscale images, this corresponds to nearly identical brightness levels. As shown in Fig. 10, the metal surface and its defects are mostly concentrated in the high-intensity region (close to white), whereas the background is primarily distributed in the low-intensity region (close to black). To strengthen the discriminative information contained in the high-intensity region, we propose a logarithm-based nonlinear gray-level transformation that redistributes the contrast in this range to achieve feature enhancement. The proposed enhancement scheme consists of two stages, logarithmic transformation and contrast stretching, whose mathematical formulations are given in Eq. (1) and Eq. (2), respectively.

Logarithmic transformation:

1 $$\:log\_img=(-1)\times\:{{log}}_{e}(1+(1-\left(\frac{img}{255}\right)\left)\right)$$

Contrast stretching:

2 $$\:new\_img=\frac{log\_img-min(log\_img)}{max\left(log\_img\right)-min(log\_img)}\times\:255$$

where $$\:img=\left[\begin{array}{ccc}{a}_{1,\:1}&\:\cdots\:&\:{a}_{1,\:n}\\\: \vdots &\:\ddots\:&\:\vdots \\\:{a}_{m,\:1}&\:\cdots\:&\:{a}_{m,\:n}\end{array}\right]$$ is the original image array, and $\:new\_img$ is the feature-enhanced image, as shown in Fig. 10.

In image processing, applying intensity transformations via nonlinear curves is a common strategy for contrast enhancement. By exploiting variations in the slope of different segments of the transformation curve, the intensity resolution can be adjusted. Regions with a steeper slope amplify the differences among pixel values within the corresponding intensity range. Among such nonlinear mappings, the logarithmic function is particularly widely used, as its slope becomes larger for input values closer to zero.

To enhance the contrast in the high-intensity regions while keeping the computation of the logarithmic function as simple as possible, the input to the logarithmic function must be preprocessed according to its characteristics. First, the original image intensities are normalized to facilitate subsequent computation and interpretation. Then, to map the originally bright regions of the image to the part of the logarithmic curve near zero, a gray-level inversion is performed after normalization.

Next, we consider the output behavior of the logarithmic function to refine the formulation of the logarithmic transform. After normalization and inversion, the input to the logarithmic function lies in the range [0, 1]. To avoid extreme values caused by arguments close to zero and to ensure strictly positive outputs, we add 1 to the input values, effectively shifting them so that a monotonic and numerically stable output is obtained. Subsequently, to restore the original gray-level polarity of the image (i.e., bright regions corresponding to larger intensity values), the logarithmic term is multiplied by a constant factor of − 1, thereby completing the contrast enhancement in the logarithmic transformation stage.

In the second stage, contrast stretching remaps the result of the nonlinear transformation back to the gray-level range [0, 255]. Specifically, we perform a linear rescaling based on the minimum and maximum values of the transformed image, so that its dynamic range is fully expanded and the brightness differences among pixel values are maximized. Through this two-stage process, subtle structures associated with defects in the high-intensity regions are magnified, while background regions remain compressed, enabling the subsequent defect detection model to focus more reliably on the critical defect features.

#### Backbone lightweight

The backbone serves as the foundational component of a neural network architecture. It employs multiple convolutional layers to extract deep features from input images and is one of the most parameter-intensive parts of the network. To achieve a lightweight design suitable for real-time detection, this study tried to replace the original YOLOv7-tiny backbone with several classical lightweight CNN architectures, thereby substantially reducing the number of network parameters while maintaining satisfactory recognition performance.

The lightweight CNN architectures considered in this work include ShuffleNetV2-0.5x to 2.0 × (2018)^16^, MobileNetV3-small/large (2019)^17^, and GhostNetV2 (2022)^18^. Using YOLOv7-tiny-Anchor-Free as the baseline, the results and a brief analysis of the backbone replacement experiments are summarized in Table 1.

**Table 1:** Analysis of backbone replacement experiment results.

Model	Parameters	mAP@0.5	mAP@0.5:0.95
YOLOv7-tiny- Anchor-Free	8,109,230	0.76073	0.48743
YOLOv7-tiny-AF- ShuffleNetV2-0.5x	5,601,486 (↓2,507,744)	0.70519 (↓0.05554)	0.45008 (↓0.03735)
YOLOv7-tiny-AF- ShuffleNetV2-1.0x	6,400,206 (↓1,709,024)	0.71021 (↓0.05052)	0.45682 (↓0.03061)
YOLOv7-tiny-AF- ShuffleNetV2-1.5x	7,521,230 (↓588,000)	0.72015 (↓0.04058)	0.46162 (↓0.02581)
YOLOv7-tiny-AF- ShuffleNetV2-2.0x	9,280,462 (↑1,171,232)	0.72738 (↓0.03335)	0.46512 (↓0.02231)
YOLOv7-tiny-AF- MobileNetV3-small	5,923,606 (↓2,185,624)	0.71362 (↓0.04711)	0.47183 (↓0.0156)
YOLOv7-tiny-AF- MobileNetV3-large	7,123,686 (↓985,544)	0.73049 (↓0.03024)	0.47911 (↓0.00832)
YOLOv7-tiny-AF- GhostNetV2	8,934,318 (↑825,088)	0.71612 (↓0.04461)	0.47524 (↓0.01219)

**Baseline Condition** Model Architecture: YOLOv7-tiny-Anchor-Free Dataset༚GC10-DET-relabel-feature-enhancement.

Dataset Split: Training Set(70%), Validation Set(20%), Test Set(10%) Epochs༚300.

Batch Size: 16.

**Fig. 11:**
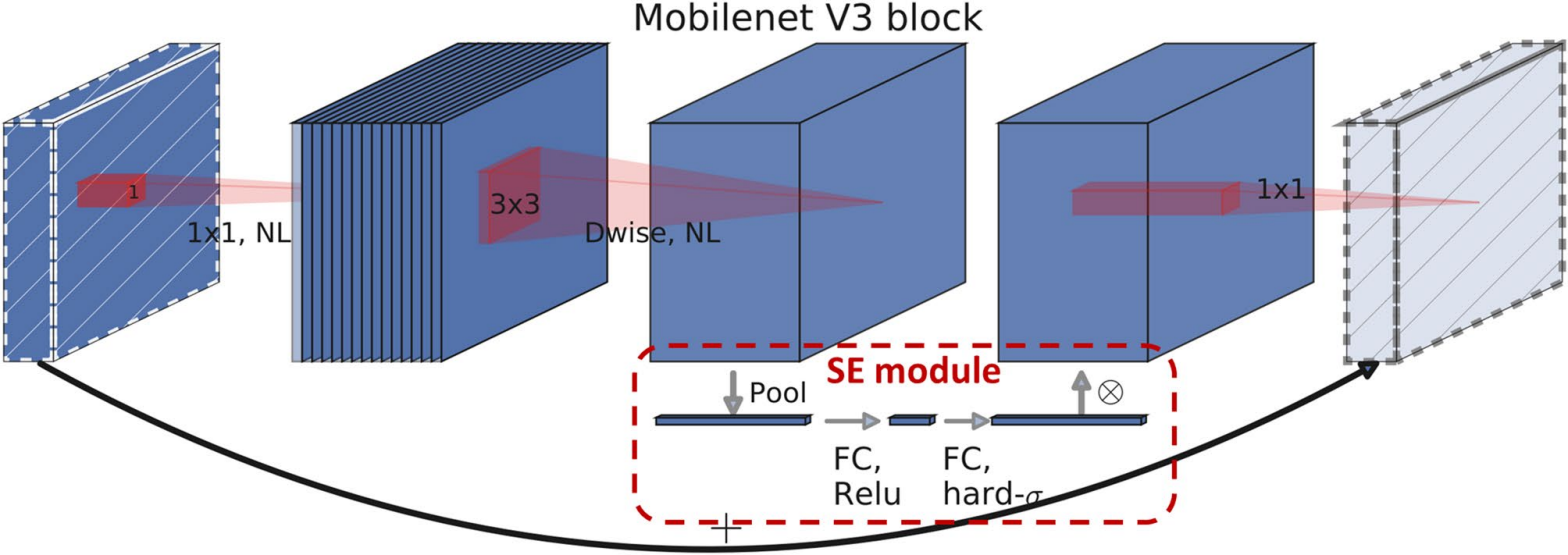
MobileNetV3 module architecture^17^.

According to the experimental results, although MobileNetV3-large is not a particularly recent architecture, its performance in metal surface defect detection surpasses that of GhostNetV2 (2022) and best satisfies the requirements of this study. When adopted as the backbone, MobileNetV3-large reduces the total number of parameters of the improved model by approximately 12%, while maintaining competitive accuracy (with mAP@0.5 decreasing by only 3% and mAP@0.5:0.95 decreasing by merely 0.8%). It is therefore selected as the main backbone architecture in this work. MobileNetV3 is a lightweight convolutional neural network (CNN) architecture proposed by Google, designed to enhance computational efficiency and performance on resource-constrained mobile and embedded devices. Its modular structure is shown in Fig. 11.

MobileNetV3 inherits the depth-wise separable convolutions from V1 and the inverted residual blocks with linear bottlenecks from V2. The former decomposes standard convolutions into depth-wise and point-wise convolutions, whose computational cost is reduced to approximately the inverse square of the kernel size. The latter improves detection performance through channel expansion and the ReLU6 activation, while reducing kernel sizes to significantly lower the computational burden. Together, these techniques markedly decrease the number of parameters and floating-point operations, while preserving high accuracy. Building on this foundation, MobileNetV3 further incorporates the Squeeze-and-Excitation (SE) module^19^ and introduces the Hard-Swish activation function, an efficient variant of Swish^20^, thereby improving both learning capacity and runtime speed. The SE module utilizes global pooling to enhance the global receptive field, while the Hard-Swish function, defined in Eq. (3), retains rich nonlinearity without involving expensive exponential operations, achieving similar performance with substantially reduced computation. The overall MobileNetV3-large network configuration is summarized in Table 2.

3 $$\:hard\:swish\left[x\right]=x\left(ReLU6\right(x+3)/6)$$

**Table 2:** MobileNetV3-large network architecture^17^.

Input	Operator	Exp Size	Out	SE	NL	s
224^2^ × 3	conv2d, 3 × 3		16		HS	2
112^2^ × 16	bneck, 3 × 3	16	16		RE	1
112^2^ × 16	bneck, 3 × 3	64	24		RE	2
56^2^ × 24	bneck, 3 × 3	72	24		RE	1
56^2^ × 24	bneck, 5 × 5	72	40	○	RE	2
28^2^ × 40	bneck, 5 × 5	120	40	○	RE	1
28^2^ × 40	bneck, 5 × 5	120	40	○	RE	1
28^2^ × 40	bneck, 3 × 3	240	80		HS	2
14^2^ × 80	bneck, 3 × 3	200	80		HS	1
14^2^ × 80	bneck, 3 × 3	184	80		HS	1
14^2^ × 80	bneck, 3 × 3	184	80		HS	1
14^2^ × 80	bneck, 3 × 3	480	112	○	HS	1
14^2^ × 112	bneck, 3 × 3	672	112	○	HS	1
14^2^ × 112	bneck, 5 × 5	672	160	○	HS	2
7^2^ × 160	bneck, 5 × 5	960	160	○	HS	1
7^2^ × 160	bneck, 5 × 5	960	160	○	HS	1
7^2^ × 160	conv2d, 1 × 1		960		HS	1
7^2^ × 960	pool, 7 × 7					1
7^2^ × 960	conv2d, 1 × 1, NBN		1280		HS	1
1^2^ × 1280	conv2d, 1 × 1, NBN					1

**Abbreviation Definitions** Input: Input Size ; Exp Size: Channel Expansion Size ; Out: Output Channel ; SE: Use of SE ; NL: Non-Linearity ; s: Stride ; bneck: Bottleneck ; NBN: No Batch Normalization ; HS: Hard-Swish ; RE: ReLU.

#### Attention module

**Fig. 12:**
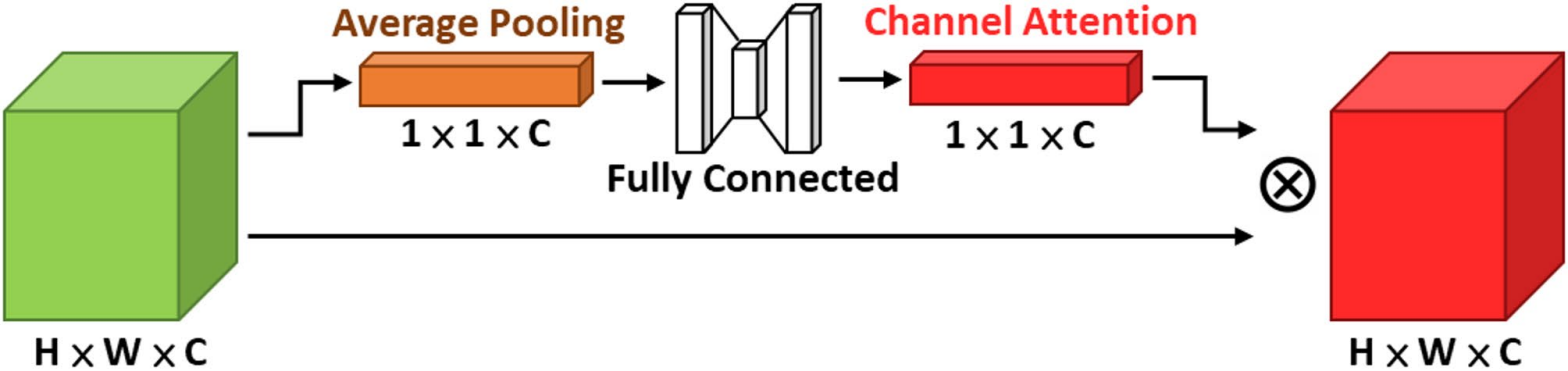
SE module architecture.

**Fig. 13:**
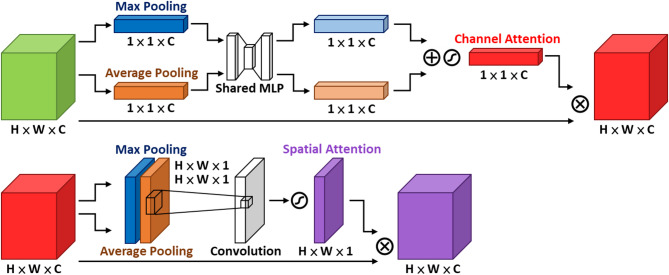
CBAM module architecture.

**Fig. 14:**
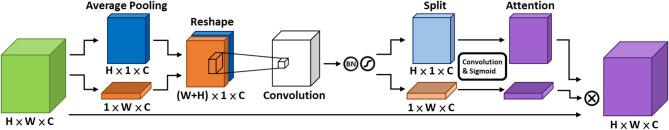
CA module architecture.

**Fig. 15:**
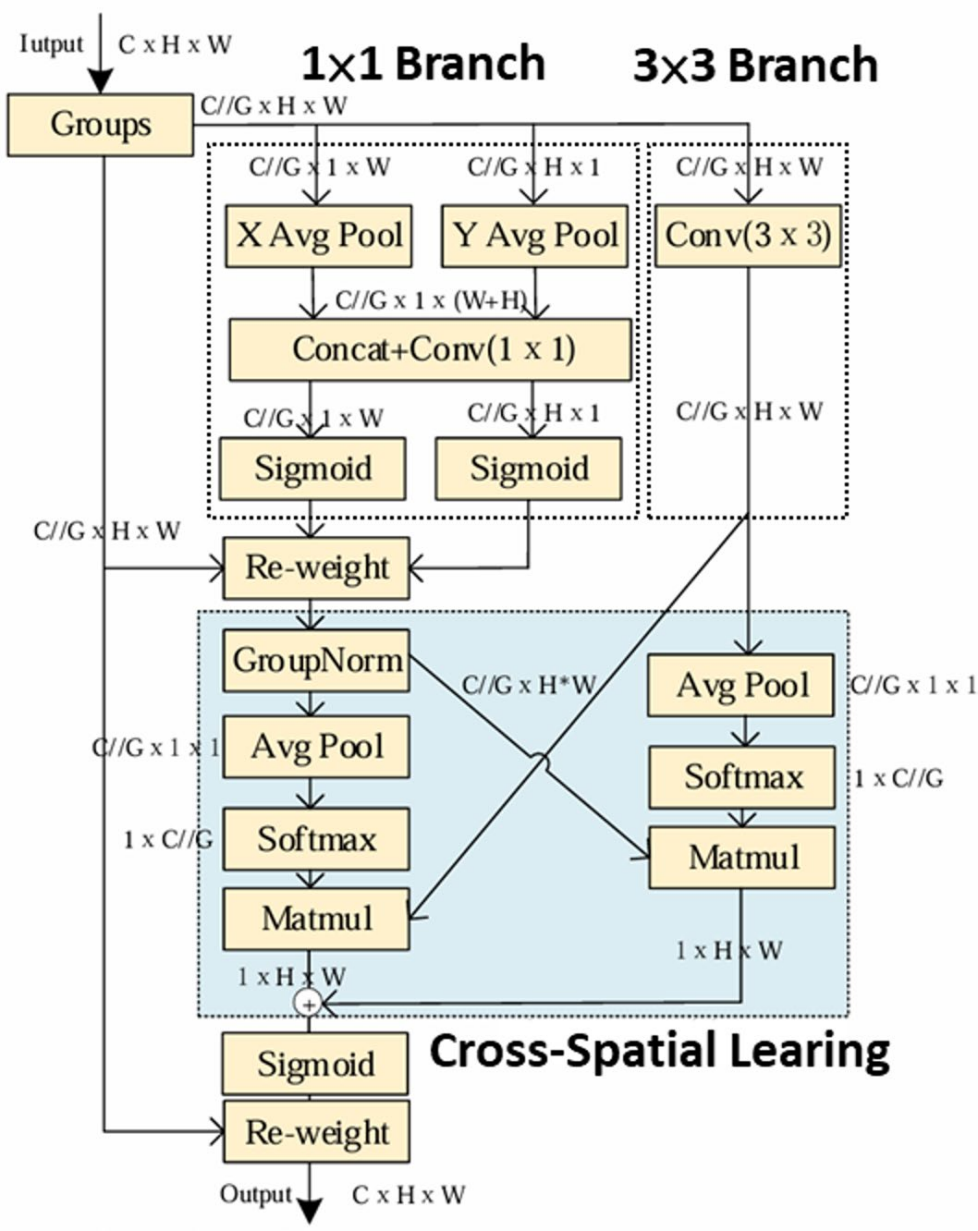
EMA module architecture^23^.

The attention mechanism is a crucial technique in contemporary machine learning and deep learning, designed to enhance model performance with only a modest increase in parameters, particularly in the recognition of small objects, where it can markedly improve discriminative capability. Representative attention modules include the SE (Squeeze-and-Excitation) module, the CBAM (Convolutional Block Attention) module^21^, the CA (Coordinate Attention) module^22^, and the EMA (Efficient Multi-Scale Attention) module^23^, as illustrated in Figs. 12, 13, 14 and 15.

The core idea can be summarized as performing specific dimensional pooling operations on the feature map to compress features, then analyzing them through convolutional layers or fully connected layers to obtain corresponding dimension weights. These weights measure the importance of elements in the feature sequence and weight the original feature map to enhance the target features’ importance and suppress background feature influence.

To compare the aforementioned CBAM, CA, and EMA modules, this study conducted a series of attention module experiments in which these three modules were individually substituted and evaluated on the complex GC10-DET dataset after annotation refinement and feature enhancement. The results are summarized in Table 3. The primary evaluation metric, fitness, follows the evaluation function proposed by the authors of YOLOv7, with particular emphasis on mAP@0.5:0.95; its specific formulation is as follows:

4 $$\:fitness=mAP@0.5\times\:0.1+mAP@0.5:0.95\times\:0.9$$

**Table 3:** Attention module replacement experiments.

Exp	Model	mAP@0.5	mAP@0.5:0.95	fitness	Parameters
(1)	YOLOv7-tiny-AF-ML-CBAM	0.7192	0.4660	0.4913	7,123,686
(2)	YOLOv7-tiny-AF-ML-CA	0.7189	0.4651	0.4904	7,042,749
(3)	YOLOv7-tiny-AF-ML-EMA	0.7345	0.4818	0.5071	7,220,112

**Abbreviation Definitions** YOLOv7-tiny-AF-ML-CBAM: YOLOv7-tiny-Anchor-Free-MobileNetV3-large-CBAM. YOLOv7-tiny-AF-ML-CA༚YOLOv7-tiny-Anchor-Free-MobileNetV3-large-CA. YOLOv7-tiny-AF-ML-E༚YOLOv7-tiny-Anchor-Free-MobileNetV3-large-EMA.

As shown in Table 3, among the three attention modules, the EMA module achieves the best detection performance on the most complex dataset used in this study. The EMA module is a novel attention mechanism derived from the CA module, proposed by Ouyang et al. in 2023, and its architecture is illustrated in Fig. 15. In their work, comparative experiments with CBAM and CA were already conducted, and the results demonstrated that EMA can enhance both image classification and object detection performance without increasing the network depth.

The EMA module first applies group convolution to partially reshape the channel dimension into the batch dimension. This grouped processing reduces computational cost while preserving fine-grained information within each group, thereby alleviating the information loss caused by pooling-based compression. Subsequently, unlike CBAM, which jointly processes channel and spatial information, EMA adopts two parallel branches to retain feature information along different dimensions. The first branch (1 × 1 branch) inherits the concept of CA and generates feature maps that are both direction-aware and position-sensitive, while the second branch (3 × 3 branch) employs a single 3 × 3 convolution with a larger receptive field to preserve precise spatial structural information. In the final stage, EMA aggregates cross-information across different spatial dimensions (Cross-Spatial Learning) to capture pixel-level pairwise relationships.

**Fig. 16:**
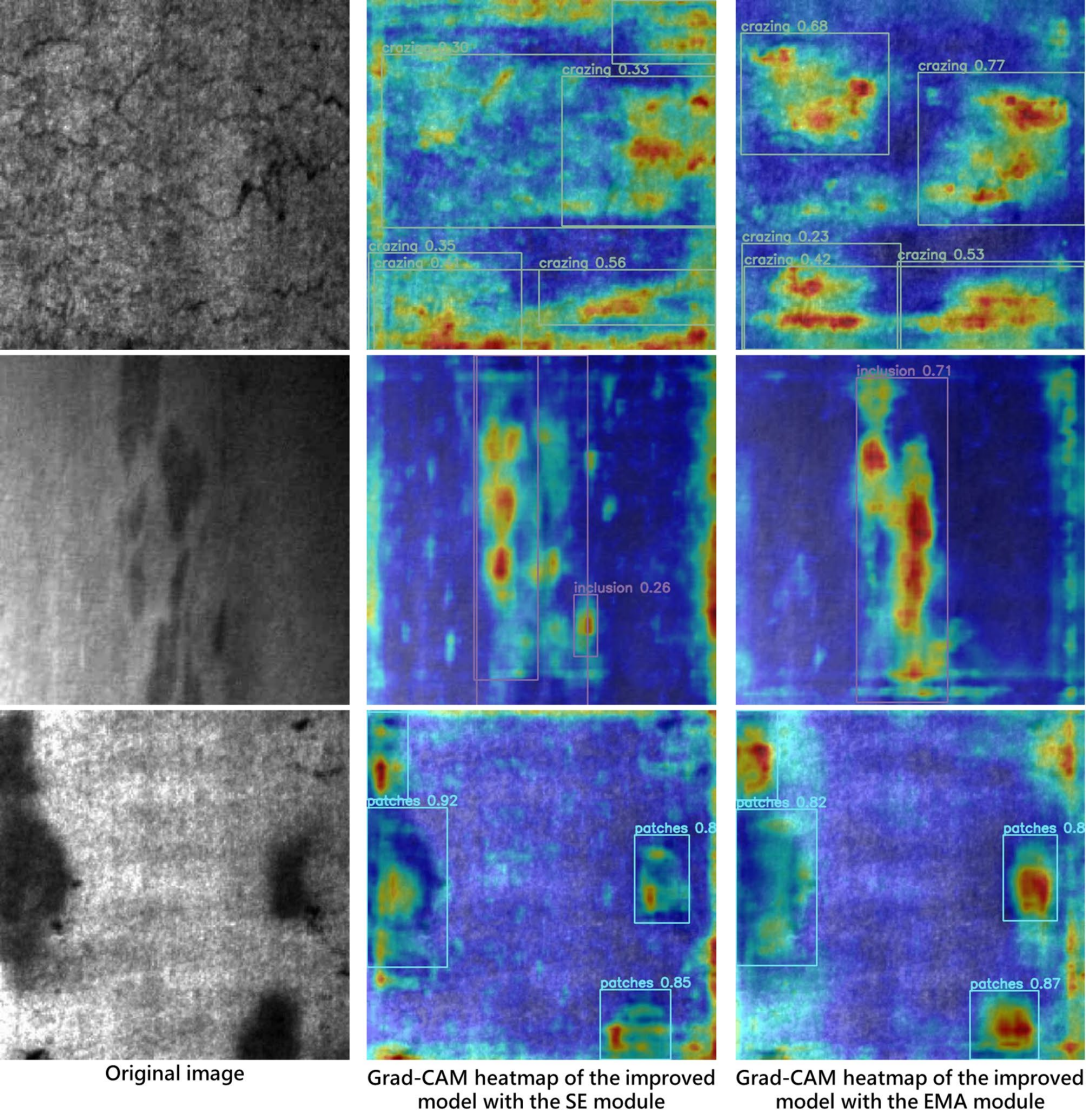
Grad-CAM heatmap of EMA module attention effect based on NEU-DET.

**Fig. 17:**
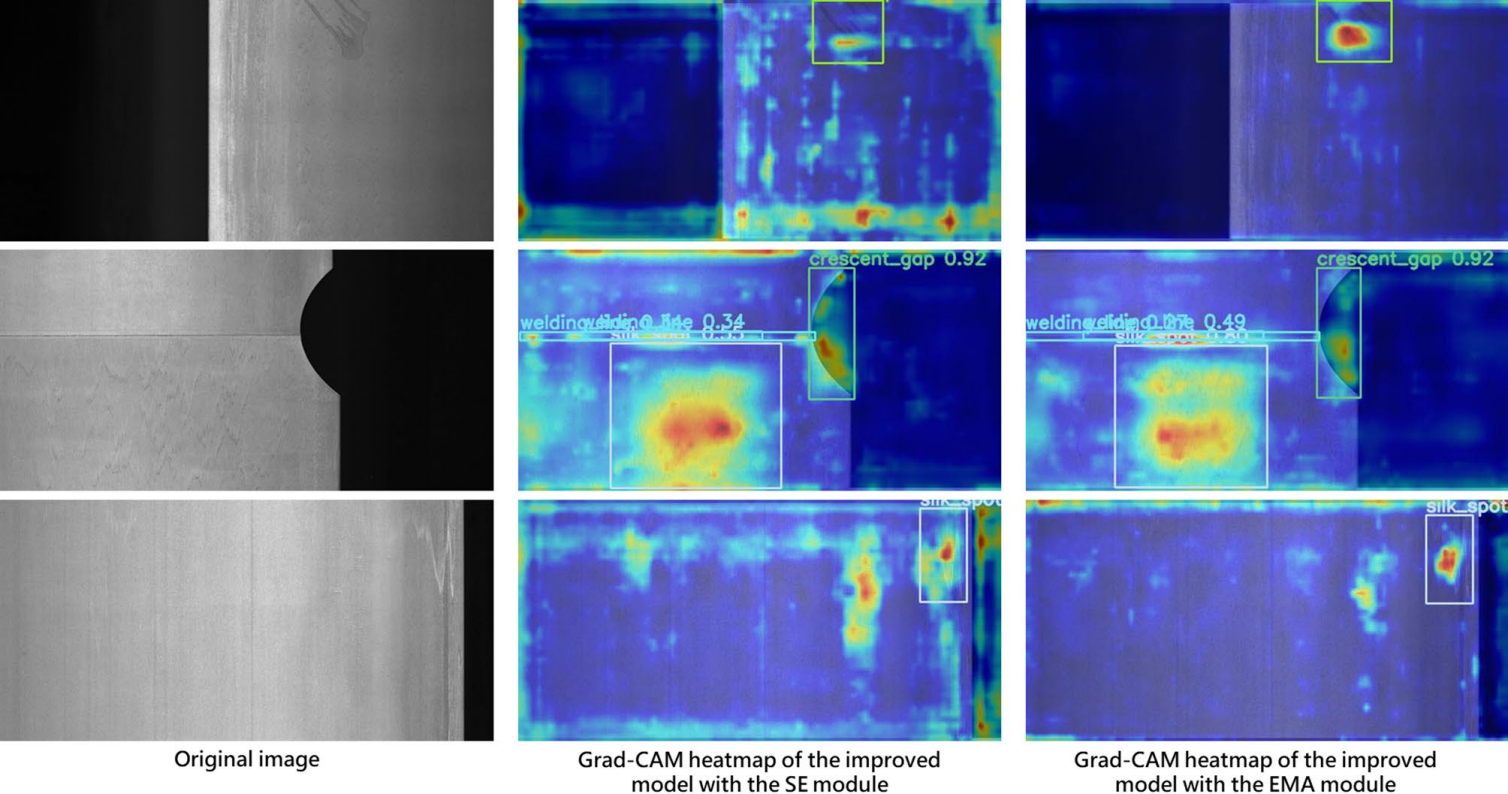
Grad-CAM heatmap of EMA module attention effect based on GC10-DET.

In this work, we replace the SE module in the MobileNetV3 bottleneck with the EMA module to improve the model’s learning capacity and convergence, particularly for the recognition of small defects. We further employ Grad-CAM heatmaps to visualize the performance differences resulting from the attention module replacement. Figures 16 and 17 present the comparative heatmaps on the NEU-DET and GC10-DET datasets, respectively. In each figure, the left image is the original input, the middle image is the heatmap generated using the default SE module in the MobileNetV3 bottleneck, and the right image is the heatmap obtained after replacing SE with the EMA attention module. It can be clearly observed that, compared with the original SE module, EMA exhibits a stronger ability to focus on salient defect regions while effectively suppressing background responses, thereby leading to a notable improvement in overall model performance.

#### BAFPN

**Fig. 18:**
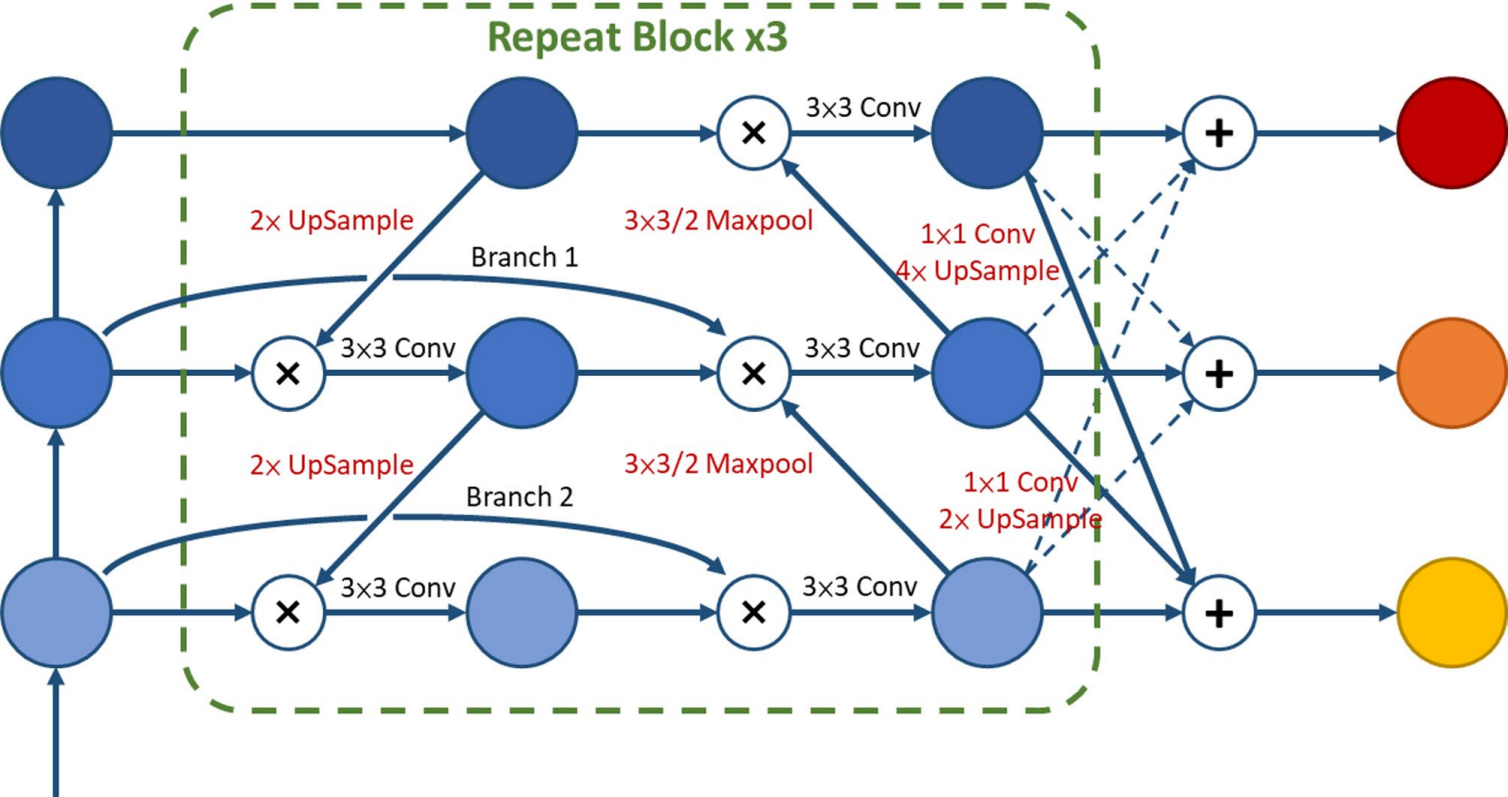
BAFPN architecture^24^.

BAFPN^24^ is a bidirectional feature pyramid network with adaptively spatial feature fusion based on PAFPN^25^, proposed by Li et al. in 2021, as illustrated in Fig. 18. Although this architecture is not among the most recent developments, it was originally introduced to enhance the performance of YOLOv4. Since YOLOv7 is an extension of YOLOv4, this study incorporates BAFPN into the proposed model modifications. Compared with the PAFPN pyramid architecture, BAFPN introduces two additional branches (Branch 1 and Branch 2 in the figure), enabling the integration of richer features without increasing computational cost. Furthermore, this structure is employed as a feature network block and is repeatedly stacked three times to strengthen feature fusion. Finally, the output features at three different scales are aggregated via upsampling and downsampling operations followed by summation, yielding new fused feature maps that provide more discriminative information for object recognition and thereby improve detection performance.

To tackle the complex texture features in metal surface defect detection, this study employs the BAFPN feature pyramid, which enhances feature fusion and integrates three-scale features, as the primary Head architecture of the improved model. To maintain a lightweight structure, we simplify the architecture by not repeatedly stacking feature network layers.

### Network training

#### Dataset configuration

**Fig. 19:**
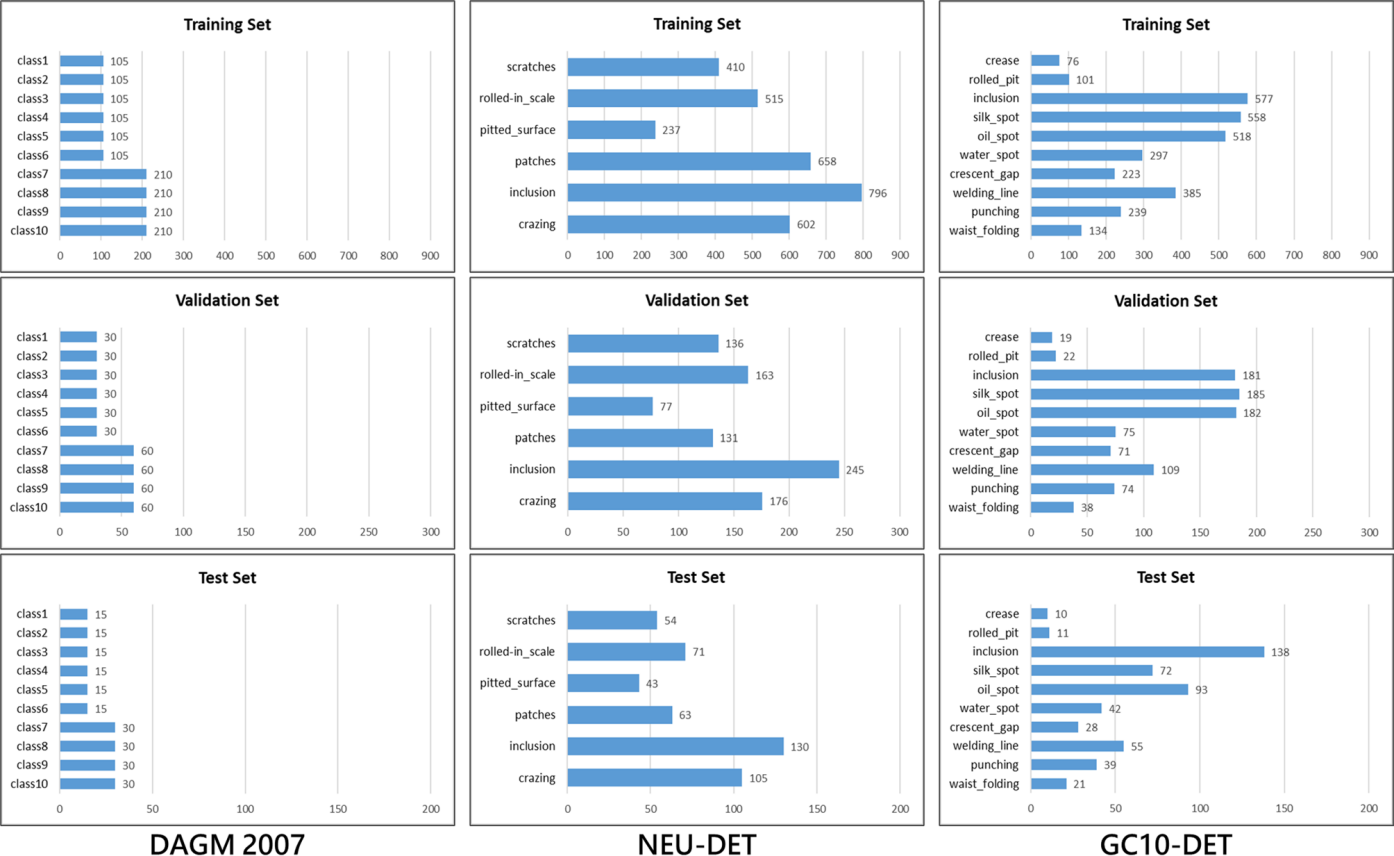
Dataset label distribution.

In this study, we divided three public datasets (DAGM 2007, NEU-DET, GC10-DET) into training sets, validation sets, and test sets in a 7:2:1 ratio. The specific data allocation is shown in Table 4, and the label distribution is illustrated in Fig. 19.

**Table 4:** Distribution of dataset sample quantities.

Dataset	Training Set Number	Validation Set Number	Test Set Number
DAGM 2007	1470	420	210
NEU-DET	1259	360	180
GC10-DET	1606	459	229

#### Equipment specifications

The experimental equipment used in this study aims to efficiently and accurately run the YOLO model and perform defect detection tasks. The hardware and software configurations of the experimental environment are as follows:

- Hardware Configuration:

1. Processor (CPU): Intel Core i7-11700 @ 2.50 GHz, 8 cores.
2. Graphics Processor (GPU): NVIDIA GeForce RTX 3080 10 GB.
3. Memory (RAM): 64 GB.

- Software Configuration:

1. Operating System: Windows 10.
2. CUDA Version: CUDA 11.7.
3. Python Version: Python 3.9.
4. Deep Learning Framework: PyTorch 1.13.
5. OpenCV Version: OpenCV 4.9.

#### Hyperparameter settings

To evaluate the effectiveness of the proposed model optimizations, all experiments were conducted under an identical computing environment with a fixed set of hyperparameters as the baseline. This design ensures that the experimental results are both reliable and comparable. The main hyperparameter configuration adopted in this study is summarized in Table 5. The training-related hyperparameters in the upper part of the table were selected with reference to commonly used settings in neural network research and were further adjusted in light of the available hardware resources. It is also worth noting that no pretrained weights were used in this work, in order to guarantee the rationality and fairness of the training process.

**Table 5:** Hyperparameter configurations.

Hyperparameter	Configuration	
Epochs	300	
Batch Size	16	
Image Size	(640, 640)	
Optimizer	SGD	
Initial Learning Rate (lr0)	0.01	
Final Learning Rate (lrf)	0.01	
	**Anchor-Base Detection Head**	**Anchor-Free Detection Head**
Localization Loss Coefficient (box)	0.05	7.5
Classification Loss Coefficient (cls)	0.5	0.3
Objectness Loss Coefficient (obj)	0.7	0.7

Regarding the weighting hyperparameters of the YOLO loss function, the official YOLOv7 settings were employed when using the original anchor-based detection head, so as to preserve the rationale behind the baseline YOLOv7-tiny configuration. For the improved anchor-free detection head, to better accommodate the direct prediction of bounding boxes, we referred to the official hyperparameter settings of YOLOv9, which likewise adopts an anchor-free head. Based on this reference, we increased the coefficient of the localization loss to emphasize the importance of bounding box (BBox) regression for the overall detection performance.

### Evaluation metrics

To evaluate the performance of the model in detecting defects on metal surfaces, this study employs performance metrics from the COCO dataset’s object detection evaluation, including Precision, Recall, Mean Average Precision (mAP), and the model’s inference speed.

**Fig. 20:**
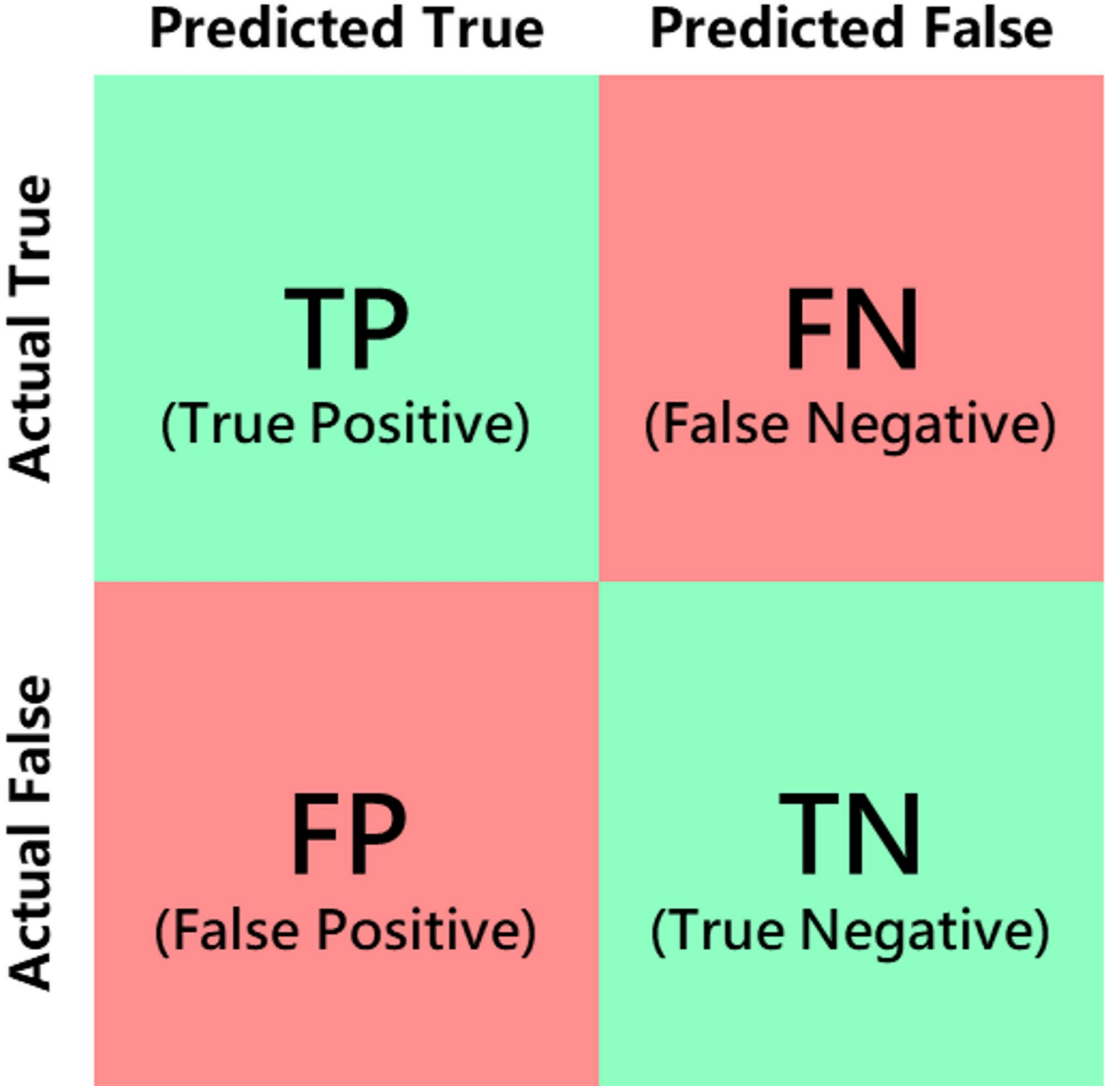
Confusion matrix.

The definitions of Precision and Recall can be clearly understood from the confusion matrix, as illustrated in Fig. 20.

In the confusion matrix, rows represent the predicted classes of the model, and columns represent the actual classes, dividing the prediction results into four types: True Positive (TP), False Negative (FN), False Positive (FP), and True Negative (TN), clearly showing the prediction situation of the model on different classes.

Corresponding to the four types in the confusion matrix, precision can be defined as the proportion of correctly predicted positive samples to all predicted positive samples, indicating the accuracy of the prediction:

5 $$\:Precision=\frac{TP}{TP+FP}$$

Recall can be defined as the proportion of correctly predicted positive samples to all actual positive samples, indicating the capture rate of the detection:

6 $$\:Recall=\frac{TP}{TP+FN}$$

With an IoU threshold of 50%, precision and recall values vary with the change in confidence threshold. Therefore, we can plot the precision and recall relationship curve (PR Curve), and the performance metric for a single class, average precision (AP), is the area under the curve:

7 $$\:AP={\int\:}_{0}^{1}P\left(R\right)dR$$

In multi-class detection, the AP metrics of each class are usually averaged to obtain the mAP, also known as mAP@0.5:

8 $$\:mAP@0.5=\frac{1}{N}{\sum\:}_{i=1}^{N}{AP}_{i}$$

Where $\:0.5$ represents the IoU threshold, $\:N$ is the total number of classes, and $\:{AP}_{i}$ is the average precision of the i-th class.

The stricter mAP@0.5:0.95 metric further considers the model’s performance at different IoU thresholds, gradually increasing from 0.5 to 0.95 with a step size of 0.05 to show the detection performance at different overlap degrees:

9 $$\:mAP@0.5:0.95=\frac{1}{10}{\sum\:}_{k=0}^{9}mAP@(0.5+0.05k)$$

Where $\:k$ is the step number of the IoU threshold, and $\:0.05$ is the step size of the IoU threshold.

### Code availability

The proposed method was implemented by extending the publicly available YOLOv9 codebase. The final model configuration and the additional module definitions required to run the final model are provided as Supplementary Software, together with a README describing how to integrate these files into the original YOLOv9 repository and reproduce the results reported in this study. Additional code materials (e.g., alternative configurations or intermediate versions used for ablation experiments) are available from the corresponding author upon reasonable request.

## Experiment

### Ablation experiments

To clearly present the entire experimental context and the impact of network improvements on model detection performance, this study used the original YOLOv7-tiny as the baseline model and the GC10-DET dataset as dataset. Through ablation experiments, we gradually added improvement components to verify the necessity of corresponding improvements. The experimental results are shown in Table 6.

**Table 6:** Ablation experiments.

Exp	Model	mAP@0.5	mAP@0.5:0.95	Parameters	GFLOPs
	Dataset				
(1)	YOLOv7-tiny	0.6541	0.3325	6,031,950	13.3
	GC10-DET				
(2)	YOLOv7-tiny-AF	0.67956	0.34953	8,109,230	21.1
	GC10-DET				
(3)	YOLOv7-tiny-AF	0.72417	0.47233	8,109,230	21.1
	GC10-DET-R				
(4)	YOLOv7-tiny-AF	0.76073	0.48743	8,109,230	21.1
	GC10-DET-R-FE				
(5)	YOLOv7-tiny-AF-ML	0.73049	0.47911	7,123,686	17.7
	GC10-DET-R-FE				
(6)	YOLOv7-tiny-AF-ML-E	0.73602	0.48155	7,220,112	23.2
	GC10-DET-R-FE				
(7)	YOLOv7-tiny-AF-ML-E-B	0.75165	0.48779	7,763,408	24.6
	GC10-DET-R-FE				

**Abbreviation Definitions** YOLOv7-tiny-AF: YOLOv7-tiny-Anchor-Free. YOLOv7-tiny-AF-ML༚YOLOv7-tiny-Anchor-Free-MobileNetV3-large. YOLOv7-tiny-AF-ML-E༚YOLOv7-tiny-Anchor-Free-MobileNetV3-large-EMA. YOLOv7-tiny-AF-ML-E-B༚YOLOv7-tiny-Anchor-Free-MobileNetV3-large-EMA-BAFPN. GC10-DET-R༚GC10-DET-relabel. GC10-DET-R-FE༚GC10-DET-relabel-feature-enhancement.

#### Experiment (1) to experiment (2)

**Fig. 21:**
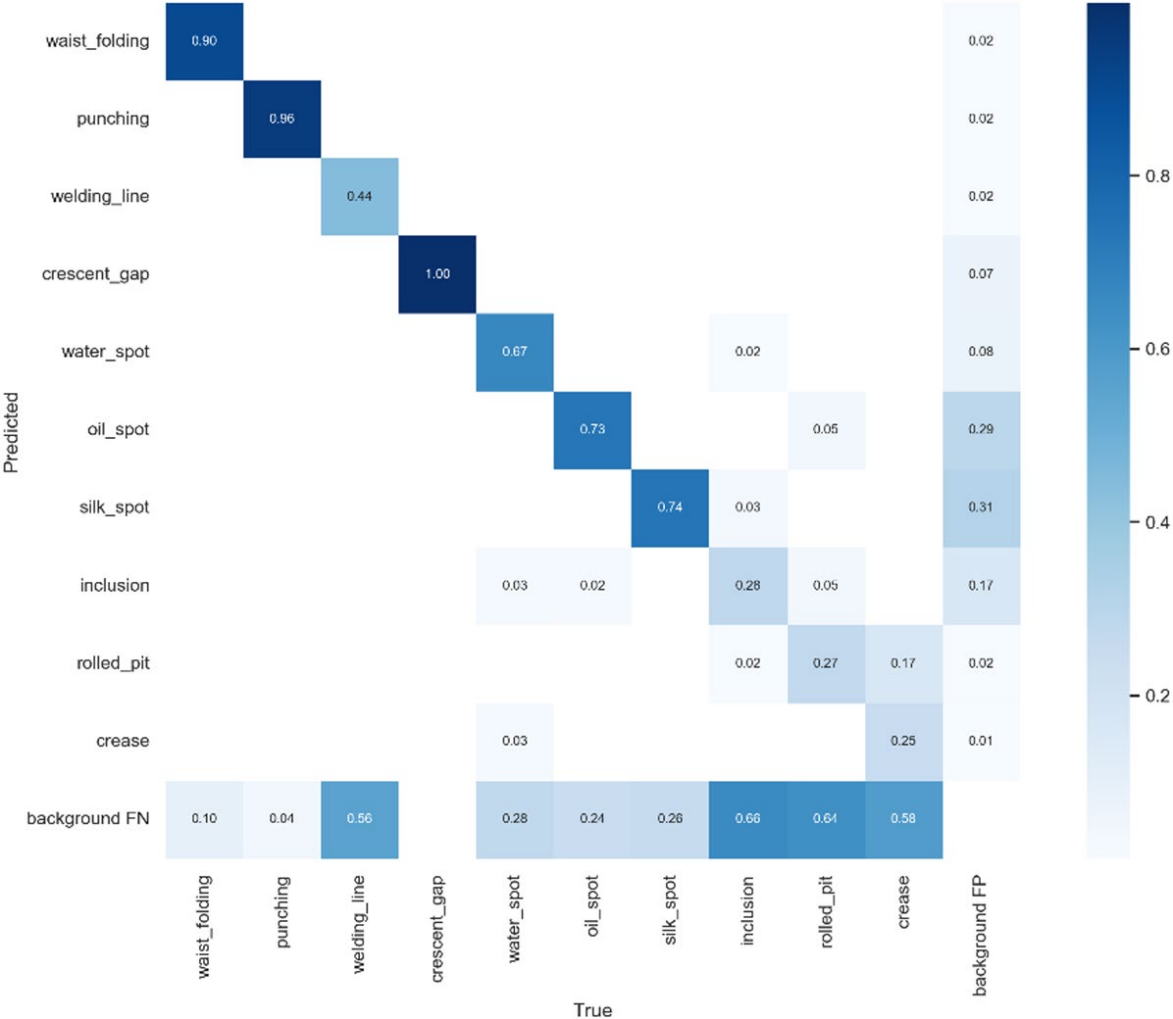
Experiment (1) confusion matrix.

From the confusion matrix of experiment (1), it is clear that the original YOLOv7-tiny model performs poorly in recognizing Weld Line, Inclusion, Rolled Pit, and Crease, as shown in Fig. 21. After analysis, we believe the reasons may be:

1. Weld Line and Crease have extreme aspect ratio linear features, which are the main reasons for poor detection performance.
2. Inclusion may be due to poor dataset annotation quality.
3. Rolled Pit and Crease may result from too few samples, leading to insufficient model generalization, as shown in Fig. 9.

**Fig. 22:**
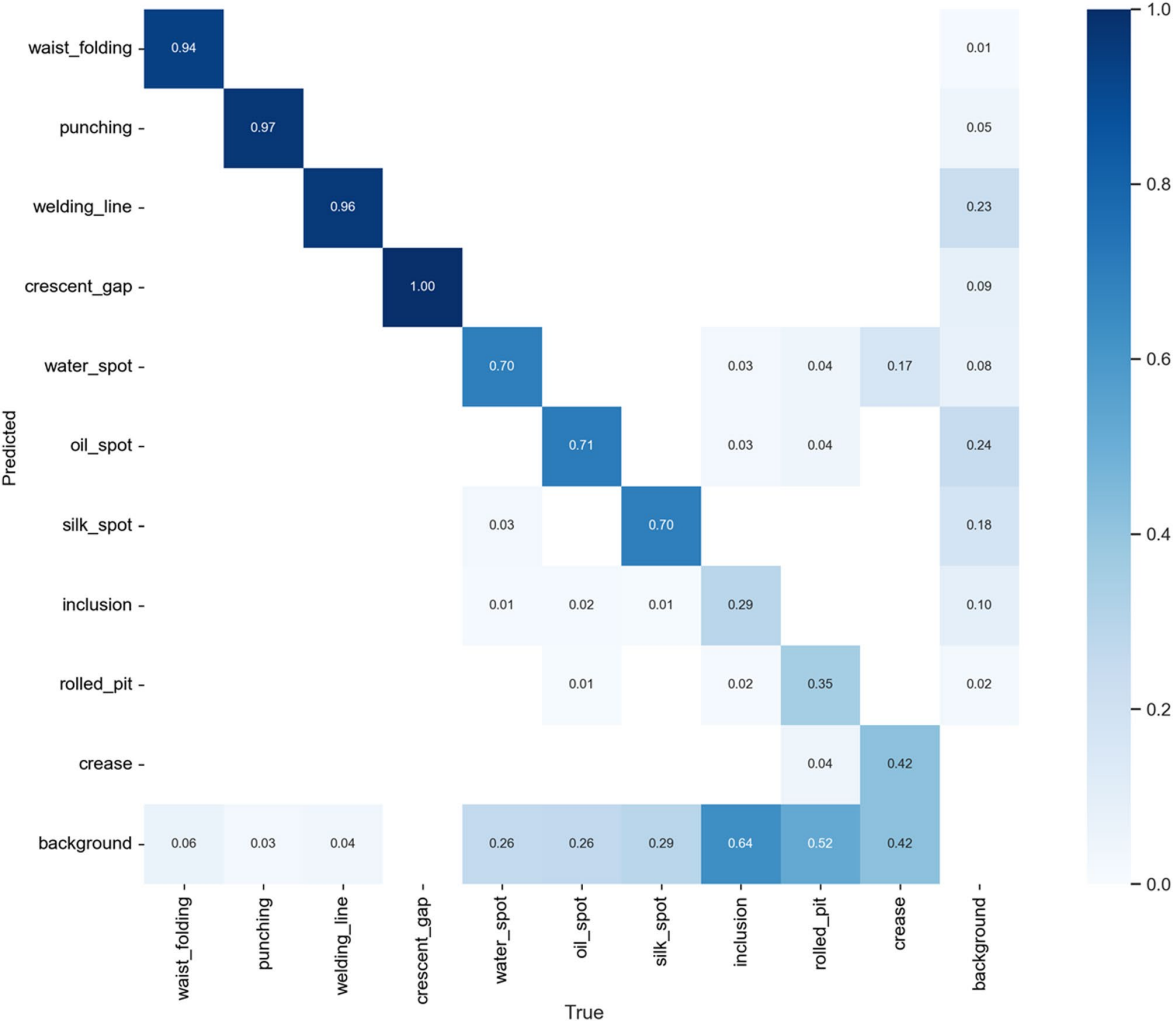
Experiment (2) confusion matrix.

For defects with extreme aspect ratios, this study improved through the replacement of the Anchor-Free detection head in experiment (2). The results, shown in Fig. 22, indicate a significant improvement in detecting Weld Line and Crease, with precision increasing by 52% and 17%, respectively.

#### Experiment (2) to experiment (3)

To solve the problem of poor dataset annotation quality, this study re-annotated the dataset. From the label statistics in Fig. 9, it can be seen that most labels increased, especially Inclusion, which increased by more than twice the data points. This approach not only improved annotation quality but also tackled some sample scarcity issues.

**Fig. 23:**
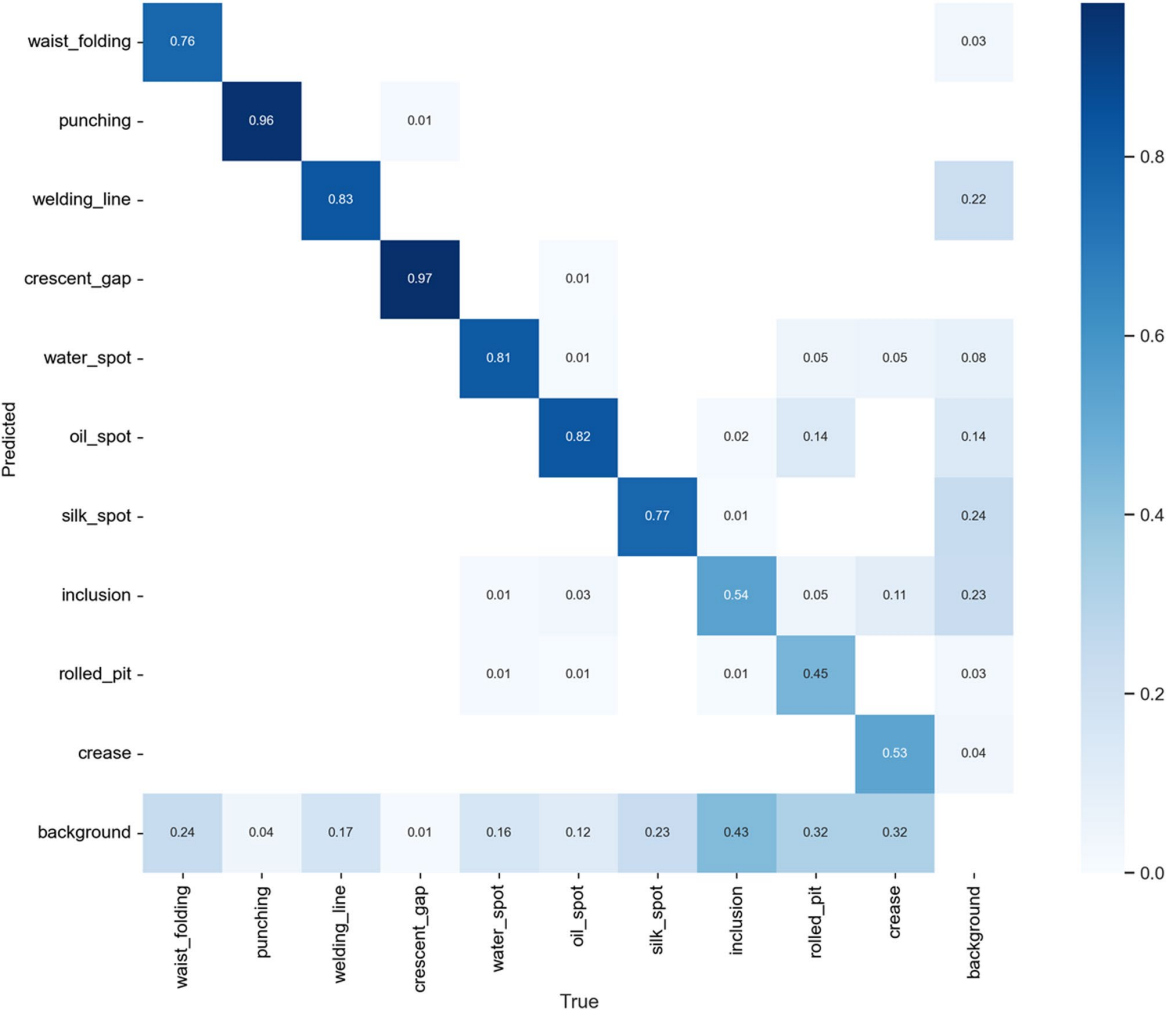
Experiment (3) confusion matrix.

The confusion matrix of experiment (3) in Fig. 23 shows that after re-annotation, the precision for Inclusion, Rolled Pit, and Crease increased by 25%, 10%, and 11%, respectively.

#### Experiment (3) to experiment (4)

**Fig. 24:**
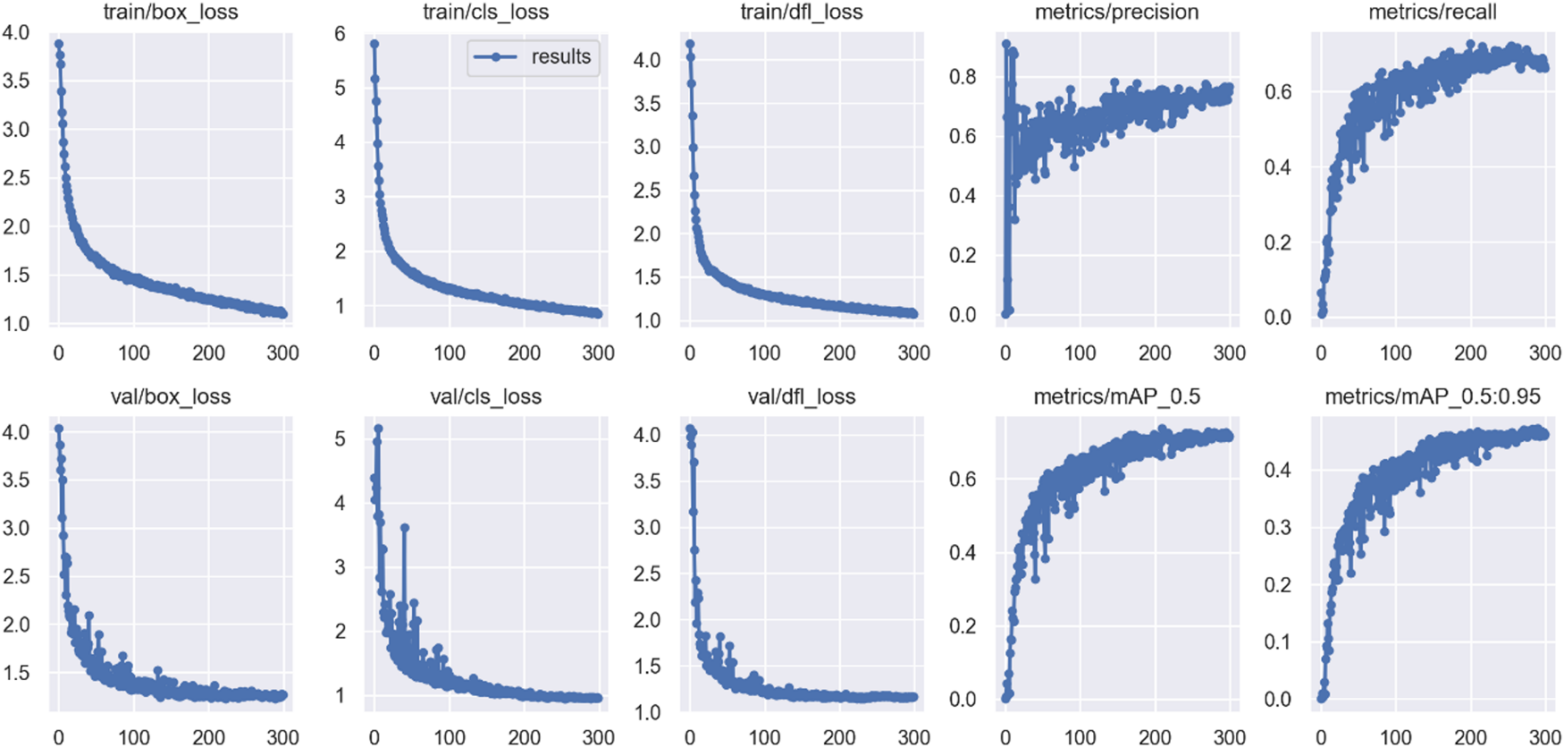
Model training metrics before feature enhancement.

**Fig. 25:**
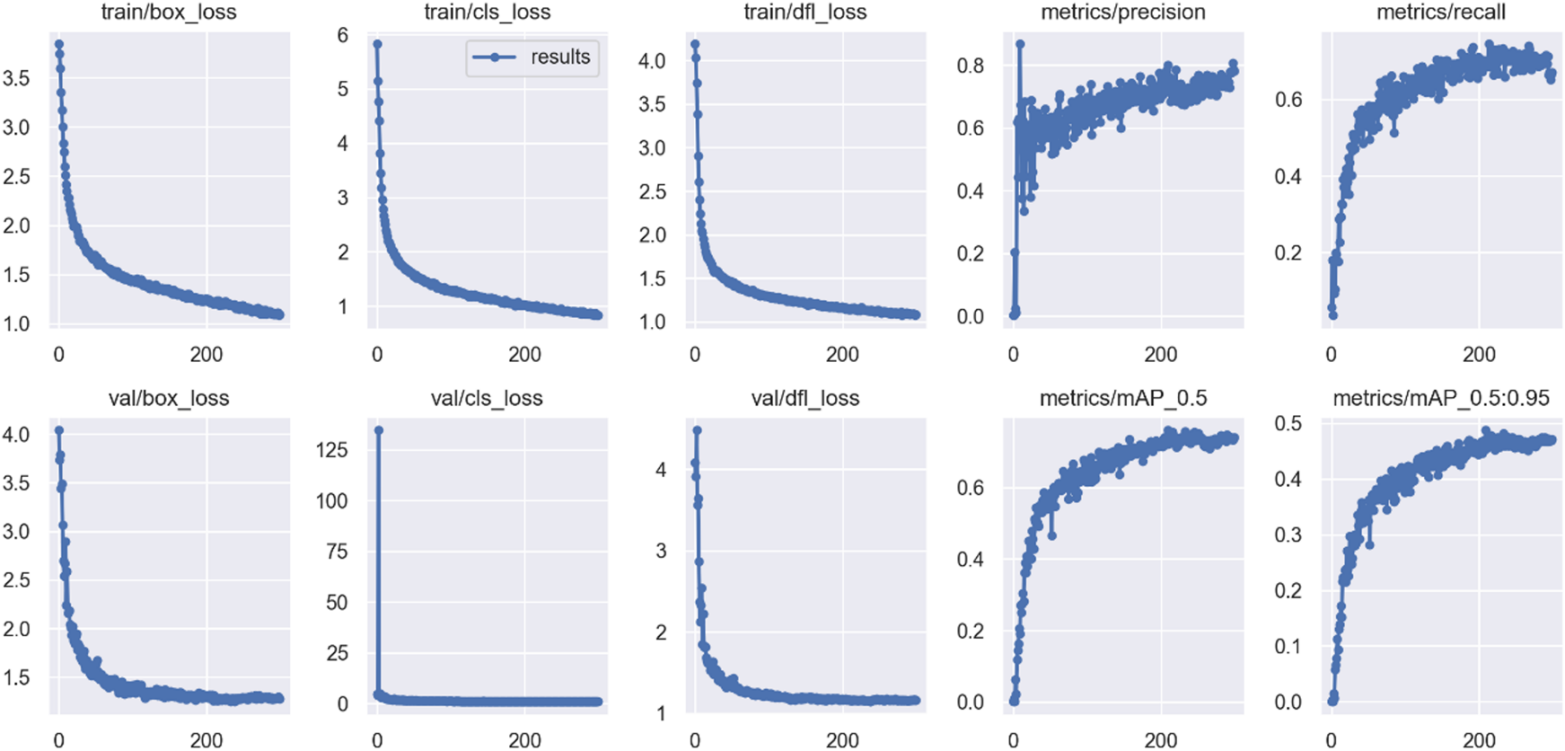
Model training metrics after feature enhancement.

To highlight the textures of defects and strengthen the differences between targets and background, this study performed feature enhancement. Figures 24 and 25 show the changes in training indicators before and after feature enhancement. After feature enhancement, the model’s convergence significantly improved, especially in validation set loss and mAP indicators, with reduced fluctuation and stable trends, indicating higher detection capability. The mAP@0.5 and mAP@0.5:0.95 indicators increased by 3.66% and 1.51%, respectively, indicating significant effects.

#### Experiment (4) to experiment (5)

**Fig. 26:**
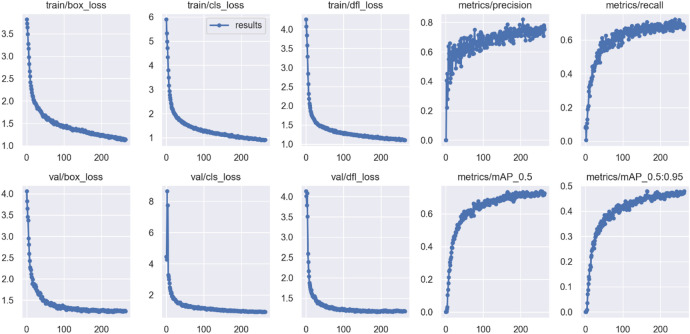
Model training metrics after backbone replacement.

To achieve lightweight, this study employed MobileNetV3-large as the backbone architecture of the model. Compared to the original backbone constructed with ELAN, it reduced the number of parameters by 985,544, accounting for 12% of the total parameters. Although there was a slight decrease in mAP metrics (mAP@0.5 decreased by 3% and mAP@0.5:0.95 decreased by only 0.8%), there was an improvement in convergence, as shown in Fig. 26.

#### Experiment (5) to experiment (6)

To enhance the detection capability for some minute defects, this study replaced the SE module in the MobileNetV3 Bottleneck architecture with the EMA module. Table 7 shows the changes in AP metrics for each category before and after adding the EMA module. The results indicate improvements in most categories, especially in Waist Folding, Water Spot, and minute defect Inclusion, with AP increases of 10.4%, 2.7%, and 3.3%, respectively.

For the overall model, mAP@0.5 increased by 0.55% and mAP@0.5:0.95 increased by 0.24%, with only a slight increase of 1.35% in the number of parameters. These results demonstrate that the EMA module successfully enhanced the model’s learning ability with minimal parameter increase.

**Table 7:** Variation in mAP for categories before and after adding the EMA module.

Class	Without EMA (AP)	With EMA (AP)	Variation
Waist Folding	0.713	0.817	↑ 0.104
Punching	0.979	0.982	↑ 0.003
Weld Line	0.848	0.757	↓ 0.091
Crescent Gap	0.992	0.994	↑ 0.002
Water Spot	0.754	0.781	↑ 0.027
Oil Spot	0.883	0.875	↓ 0.008
Silk Spot	0.722	0.727	↑ 0.005
Inclusion	0.401	0.434	↑ 0.033
Rolled Pit	0.580	0.572	↓ 0.008
Crescent Gap	0.423	0.423	-

#### Experiment (6) to experiment (7)

To tackle the high complexity texture features of metal surface defect detection, this study adopted the BAFPN architecture, integrating three-scale feature outputs, as the main Head architecture of the improved model to enhance feature fusion and improve detection performance. The final improved model achieved mAP@0.5 and mAP@0.5:0.95 indicators of 75.17% and 48.78%, respectively, indicating overall improvement effects.

### Comparative experiments

In the same computational environment and with the same hyperparameter baseline, this study used three public datasets (DAGM 2007, NEU-DET, GC10-DET) to compare the performance of the YOLOv7-tiny model and the improved model to verify the effectiveness and generalization of the proposed method.

Table 8 shows the performance comparison of the YOLOv7-tiny model and the improved model. The improved model’s performance significantly improved on the DAGM 2007, NEU-DET, and GC10-DET datasets. The mAP@0.5 indicators increased by 0.73%, 8.22%, and 9.76%, respectively, and the mAP@0.5:0.95 indicators increased by 6.42%, 17.72%, and 15.53%. Additionally, there were significant optimization effects in precision and recall.

**Table 8:** Comparison of performance metrics between YOLOv7-tiny and improved model.

Exp		Model	mAP@0.5	mAP@0.5:0.95	Precision	Recall
		Dataset				
(1)	Before Optimization	YOLOv7-tiny	0.9802	0.6294	0.9332	0.9917
		DAGM 2007				
	After Optimization	Improved Model	0.98753	0.69363	0.97214	0.96957
		DAGM 2007				
(2)	Before Optimization	YOLOv7-tiny	0.693	0.3539	0.5892	0.7119
		NEU-DET				
	After Optimization	Improved Model	0.77517	0.53111	0.7399	0.74618
		NEU-DET-R-FE				
(3)	Before Optimization	YOLOv7-tiny	0.6541	0.3325	0.746	0.5855
		GC10-DET				
	After Optimization	Improved Model	0.75165	0.48779	0.77777	0.69226
		GC10-DET-R-FE				

**Abbreviation Definitions** Improved Model: YOLOv7-tiny-Anchor-Free-MobileNetV3-large-EMA-BAFPN. NEU-DET-R-FE: NEU-DET-relabel-feature-enhancement. GC10-DET-R-FE: GC10-DET-relabel-feature-enhancement.

### Test set validation

This study evaluates the performance of the improved model using the clean (primary), third-party test set to ensure the fairness of the training results. This result aims to demonstrate that the outcome of developed high-level performance is not due to overfitting. Still, it provides an objective assessment of the model’s actual performance and tests the generalization ability of the improved model. The results are shown in Table 9.

**Table 9:** Performance metrics of the improved model on the test dataset.

Exp	Model	mAP@0.5	mAP@0.5:0.95	Precision	Recall
	Dataset				
(1)	Improved Model	0.99	0.723	0.97	0.987
	DAGM 2007				
(2)	Improved Model	0.748	0.506	0.739	0.702
	NEU-DET-R-FE				
(3)	Improved Model	0.769	0.478	0.817	0.699
	GC10-DET-R-FE				

**Abbreviation Definitions** Improved Model: YOLOv7-tiny-Anchor-Free-MobileNetV3-large-EMA-BAFPN.

NEU-DET-R-FE: NEU-DET-relabel-feature-enhancement. GC10-DET-R-FE: GC10-DET-relabel-feature-enhancement.

Comparing Table 9 with Table 8 shows that the improved model performed well on both validation and test sets, with small fluctuations in various indicators. On the three datasets, the mAP@0.5 indicators fluctuated within ± 0.25%, ± 2.72%, and ± 1.74%, respectively, while the mAP@0.5:0.95 indicators fluctuated within ± 2.94%, ± 2.51%, and ± 0.98%, confirming that the excellent performance was not due to overfitting.

To test whether the improved model meets real-time detection requirements, we measured its inference time and converted it into FPS for evaluation. The results are shown in Table 10.

**Table 10:** FPS evaluation experiment.

Exp	Model	Inference Time (ms)	FPS
	Dataset		
(1)	Improved Model	10.6	94
	DAGM 2007		
(2)	Improved Model	6.6	151
	NEU-DET-R-FE		
(3)	Improved Model	11.0	90
	GC10-DET-R-FE		

**Abbreviation Definitions** Improved Model: YOLOv7-tiny-Anchor-Free-MobileNetV3-large-EMA-BAFPN.

NEU-DET-R-FE: NEU-DET-relabel-feature-enhancement. GC10-DET-R-FE: GC10-DET-relabel-feature-enhancement. Note: The FPS calculation only considers the inference process and does not include image display. In actual use, FPS will be limited by image output, as well as the specifications of the device and camera.

The data in Table 10 shows that the improved model has extremely high inference speed, with FPS exceeding 90, meeting the real-time detection requirements of this study.

### SOTA (state-of-the-art) models comparison

To investigate and compare the proposed improved model with other SOTA models, multiple detectors were trained and evaluated on our revised GC10-DET dataset, with the detailed quantitative results summarized in the Table 11.

**Table 11:** Comparison of SOTA models by our improved GC-DET dataset.

Exp	Model	mAP@0.5	mAP@0.5:0.95	fitness	Parameters	GFLOPs
(1)	YOLOv8	0.724	0.457	0.484	3,012,798	8.2
(2)	YOLOv10s	0.722	0.482	0.506	8,074,092	24.8
(3)	YOLO11	0.704	0.452	0.476	2,591,790	6.5
(4)	YOLO12	0.706	0.459	0.483	2,569,998	6.5
(5)	Improved Model	0.769	0.478	0.507	7,763,408	24.6

**Abbreviation Definitions** Improved Model: YOLOv7-tiny-Anchor-Free-MobileNetV3-large-EMA-BAFPN.

The data show that the proposed improved model attains the highest fitness score. Although the difference from the closest competitor, YOLOv10s, is not substantial, achieving a higher mAP with lower GFLOPs is already nontrivial. A closer inspection reveals that the two models achieve comparable mAP@0.5:0.95, whereas, in terms of mAP@0.5, the proposed model exceeds YOLOv10s by nearly 5% points (0.05), clearly outperforming the other models.

Although the GFLOPs of the proposed model are considerably higher than those of YOLOv8, YOLO11, and YOLO12, these three smaller models exhibit noticeably limited overall performance. By contrast, YOLOv10s, whose GFLOPs are closer to our model, achieves results that are much more comparable. This suggests that improving performance on the metallic surface defect detection inherently requires additional computational cost. For industries that demand high-quality steel plates, however, maximizing the detection of defects is particularly critical; as long as the required FPS for online inspection is satisfied, an increase in computational cost is acceptable.

In the comparison Table 12, all state-of-the-art models are trained on the dataset adjusted in this study; therefore, the Table 12 alone cannot reveal the separate contributions of our dataset relabeling strategy and feature enhancement method. To further analyze these effects, we compare our results with those reported in the research that done by Wu^26^ et al., which also employed the GC10-DET dataset and provided a consolidated benchmark of several SOTA models. In this work, we directly adopt their reported performance figures to conduct a multi-model comparison and examine the joint improvements achieved through our dataset refinement and model modifications. As shown in the Table 12, our method yields substantially higher performance than the other models, and the magnitude of improvement is consistent with the trends observed in the preceding ablation experiments (Table 6). These results provide additional validation of the rationality and effectiveness of the proposed approach for metallic surface defect detection.

**Table 12:** Comparison of SOTA models by GC-DET dataset^26^.

ss	mAP@0.5
YOLOv5	62.5
YOLOv8	63.3
YOLOv9	64.4
YOLOv10	60.0
YOLOv11	64.2
Faster R-CNN	58.9
SAM-DETR	64.5
DAB-DETR	64.8
DN-DETR	64.9
PMSA-DETR	65.0
SH-DETR	65.0
Ours	76.9

**Fig. 27:**
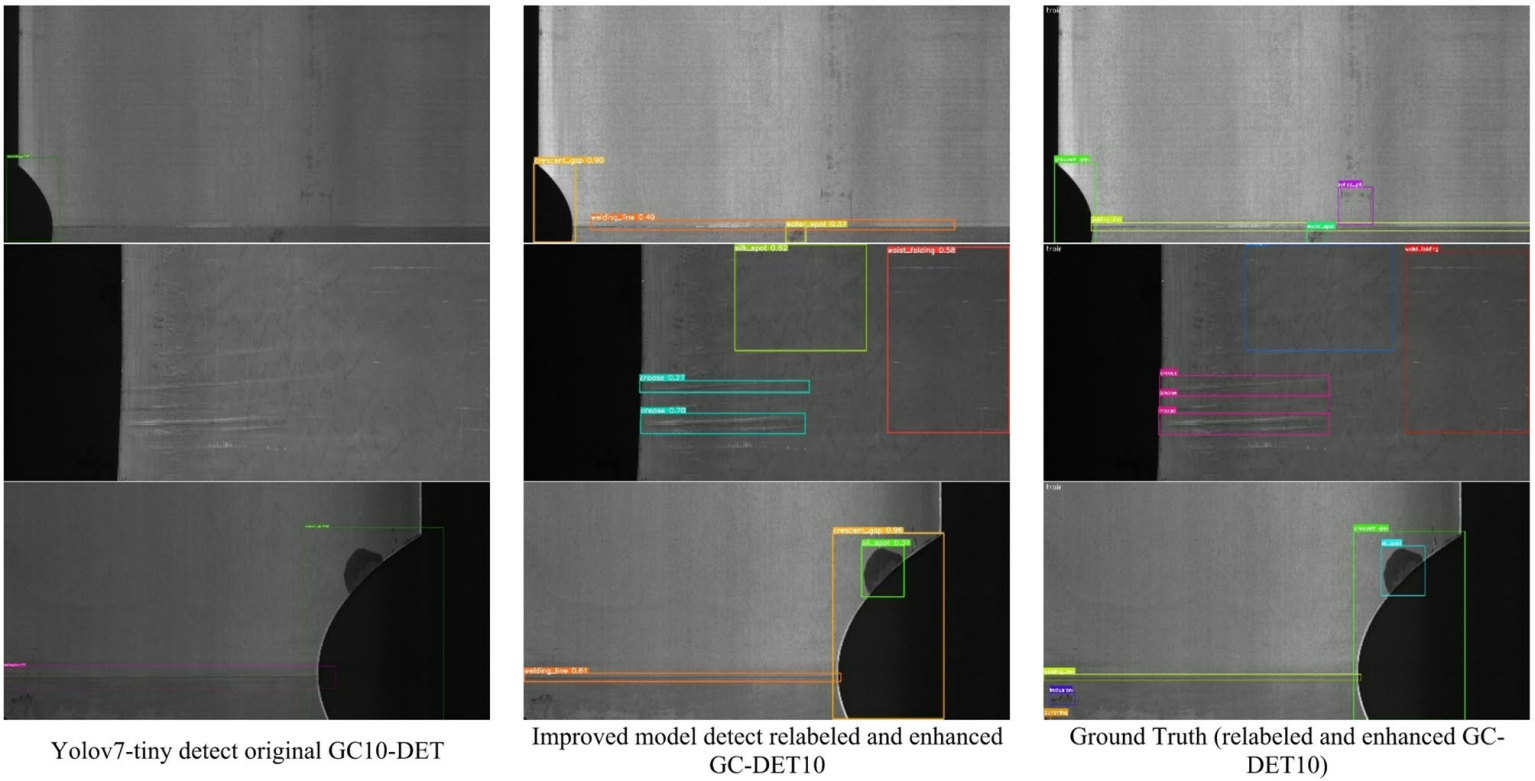
Detection results comparison between YOLO and the proposed enhanced model.

Finally, we present results in the Fig. 27 to visually and intuitively illustrate the performance of the proposed method.

## Conclusion

In this study, we propose a novel modification method for the YOLO-tiny model architecture to improve the challenges encountered in industrial metal surface defect detection:

1. To improve the detection of defects with extreme aspect ratios, the original Anchor-Base detection head is replaced with an Anchor-Free method to overcome the limitations of Anchor Boxes.
2. To enhance the feature representation of metal surface defects, feature enhancement processing is applied to the data. This is based on a logarithmic transformation, which amplifies the intervals in the bright areas of the image while compressing the intervals in the dark areas.
3. To achieve the goal of lightweight real-time detection, MobileNetV3-large is used as the backbone architecture of the improved model, reducing the total number of parameters by approximately 12%.
4. To improve model performance, especially for minute target detection, we replaced the SE module in the MobileNetV3 Bottleneck architecture with the EMA attention module to enhance target attention and suppress background features.
5. To overcome the highly complex texture features in metal surface defect detection, the BAFPN bidirectional feature pyramid architecture, which integrates three-scale feature outputs, is used as the main Head architecture. This provides more feature information for recognition, thereby improving detection performance.

We compared the detection results on three metal surface defect datasets to verify the effectiveness and generalization of the proposed method. The experimental data showed significant detection accuracy improvements for the three datasets of different complexity levels. On the DAGM 2007 dataset, the mAP@0.5 increased from 98.02% to 98.75%, and the mAP@0.5:0.95 increased from 62.94% to 69.36%. On the NEU-DET dataset, the mAP@0.5 increased from 69.30% to 77.52%, and the mAP@0.5:0.95 increased from 35.39% to 53.11%. On the GC10-DET dataset, the mAP@0.5 increased from 65.41% to 75.17%, and the mAP@0.5:0.95 increased from 33.25% to 48.78%. In terms of model training, the improved model showed better convergence by observing training indicator charts. In terms of detection speed, the inference process FPS exceeded 90, meeting the real-time computing requirements of metal surface defect detection.

Building upon the insights gained from this study, we outline several promising directions for future works:

1. Hyperparameter settings significantly impact model performance. Using fixed hyperparameters as the baseline ensures fairness but may obscure some upper limits of model accuracy. In the future, hyperparameter optimization techniques such as Bayesian Optimization (BO) or Genetic Algorithm (GA) can be used to optimize hyperparameters to fully unleash model performance.
2. The datasets used in this study, NEU-DET and GC10-DET, have some imbalance issues in the number of samples, leading to insufficient generalization for some classes. To solve this problem, Generative Adversarial Network (GAN) can be used for data augmentation in the future to solve the issue of insufficient samples.
3. This study used three public datasets to research and verify metal surface defect detection, but it still lacks the opportunity for practical verification. In the future, if possible, cooperation with related industries can be attempted for practical verification in actual environments.

## Data Availability

The datasets used and/or analysed during the current study are available from the corresponding author on reasonable request. Or the datasets can be accessed directly fromhttps://ychlab8.wixsite.com/ychlab/s-projects-side-by-side.

## Declarations

### Reprints and permisssions information

is available at www.nature.com/reprints.
